# Global, regional, and national trends in blindness and vision loss, 1990–2021: a secondary ecological trend analysis based on modelled population estimates

**DOI:** 10.7189/jogh.16.04176

**Published:** 2026-05-29

**Authors:** Shi-Nan Wu, Yuqi Jiang, Wenying Guan, Changsheng Xu, De-Xing Zeng, Bing Yan, Jingyao Lv, Caihong Huang, Jiaoyue Hu, Yi Han, Zuguo Liu

**Affiliations:** 1 Xiamen University affiliated Xiamen Eye Center; Fujian Provincial Key Laboratory of Ophthalmology and Visual Science; Fujian Engineering and Research Center of Eye Regenerative Medicine; Eye Institute of Xiamen University; School of Medicine, Xiamen University, Xiamen, Fujian, China; 2 School of Medicine, Xiamen University, Xiamen, China; 3 Department of Ophthalmology, Xiang'an Hospital of Xiamen University, Xiamen, Fujian, China; 4 Department of Ophthalmology, The First Affiliated Hospital of University of South China, Hengyang, Hunan, China

## Abstract

**Background:**

We aimed to provide global, regional, and national estimates of the burden of blindness and vision loss from 1990 to 2021, stratified by cause, age, and sociodemographic index (SDI).

**Methods:**

This population-based study used data from the 2021 Global Burden of Diseases, Injuries, and Risk Factors Study 2021 (GBD) study, covering 1990 to 2021. It evaluates the burden of blindness and vision loss based on prevalence, age-standardised prevalence rate (ASPR), disability-adjusted life years (DALYs), age-standardised DALY rate (ASR of DALY), and average annual percent changes (AAPCs).

**Results:**

Globally, the ASPR of blindness and vision loss increased from 12 453.52 (95% uncertainty interval (UI) = 10 287.58, 15 226.09) in 1990 to 15 784.33 (95% UI = 12 761.44, 19 502.32) in 2021, with an AAPC of 0.85 (95% confidence interval (CI) = 0.7, 1.11, *P* < 0.001). In contrast, the ASR of DALYs remained stable, changing from 342.01 (95% UI = 237.87, 482.17) in 1990 to 342.78 (95% UI = 224.21, 503.61) in 2021, with an AAPC of 0.05 (95% CI = −0.04, 0.2, *P* = 0.298). Notably, near vision loss and cataract showed consistent increases, with AAPCs of 1.14 (95% CI = 0.94, 1.51, *P* < 0.001) and 0.25 (95% CI = 0.16, 0.33, *P* < 0.001), respectively. Blindness and vision loss prevalence was significantly higher in low and low-middle SDI regions compared to other SDI regions.

**Conclusions:**

Global blindness and vision loss increased from 1990 to 2021, while DALYs stayed stable. Prevention should be tailored by region, country, gender, cause, and age.

With global improvements in sociodemographic status and life expectancy, the burden of disease has progressively shifted from infectious diseases to non-communicable diseases and disabilities, with vision impairment emerging as a major global public health challenge [1]. The leading causes of vision impairment include refractive disorders, cataracts, glaucoma, age-related macular degeneration, near vision loss, and other ocular conditions [2]. According to the Global Burden of Disease (GBD) 2019 study, an estimated 41.91 million people worldwide are blind, 253.08 million have moderate vision impairment, and 33.78 million have severe vision impairment, with a substantial burden also observed among children [3,4]. The World Health Organization has further highlighted that at least 2.2 billion people globally are affected by vision impairment or blindness, of whom nearly one billion cases are preventable or remain unaddressed [5]. These figures underscore the urgent need for precise, up-to-date, and cause-specific assessments to inform global eye health policies, resource allocation, and health care planning [6].

Previous large-scale efforts, including those by the Vision Loss Expert Group and earlier GBD studies, have provided foundational estimates of the global burden of blindness and vision loss, with recent updates extending to 2020 [7]. However, several important limitations remain. Prior analyses often relied on relatively coarse stratification, including broad age categories, aggregated regional classifications, and limited integration with sociodemographic index (SDI) gradients [8–10]. In addition, few studies have incorporated long-term projections with scenario-based modeling. Moreover, comprehensive evaluations of cause-specific disparities across gender, age, SDI, and geography using the most recent post-pandemic GBD 2021 data remain lacking.

To address these gaps, we conducted a global, regional, and national analysis of blindness and vision loss from 1990 to 2021 using the GBD 2021 database. We systematically examined temporal trends, cause-specific prevalence, and disability-adjusted life year (DALY) rates, with stratification by gender and detailed age groups. This study advances prior work in four key aspects:

(1) applying Joinpoint regression to identify trend inflection points and Bayesian Age-Period-Cohort (BAPC) modelling for projections to 2050, alongside adherence to the Guidelines for Reporting Analyses of Big Data Repositories Open to Public (GRABDROP) [11];

(2) performing granular stratification by 5-year age intervals, five SDI levels, 204 countries, 26 regions, and six specific causes, with cross-stratification by age and sex;

(3) quantifying the relationship between SDI and age-standardised prevalence and DALY rates, and identifying divergent trends across causes;

(4) generating scenario-based forecasts to inform future policy and planning.

Overall, this study provides an up-to-date, comprehensive, and methodologically robust assessment of the global burden of blindness and vision loss. By elucidating spatiotemporal trends, socioeconomic disparities, and interactions across age, sex, and causes, our findings offer actionable evidence to support targeted prevention strategies, optimise resource allocation, and guide global eye health policy beyond prior incremental updates.

**Adherence to JoGH’s Guidelines for Reporting Analyses of Big Data Repositories Open to the Public (GRABDROP)**

Our study complies with the Journal of Global Health (JoGH) GRABDROP [11] as detailed in Table S1 of the **Online Supplementary Document**.

## METHODS

### Data sources

The burden data for blindness and vision loss were obtained from the GBD 2021 study. These data encompass diseases classified under the International Classification of Diseases, 10th Revision (ICD-10) codes H25-H28.8, H31-H36.8, H40-H40.9, H42-H42.8, and H46-H54.9, including age-related macular degeneration (ICD-10: H35.3–H35.389), cataract (ICD-10: H25–H26.9, H28–H28.8), glaucoma (ICD-10: H40–H40.9, H42–H42.8), near vision loss and refraction disorders (ICD-10: H52–H52.7), and other vision loss (ICD-10: H27–H27.9, H31–H35.23, H35.4–H36.8, H46–H51.9, H53–H54.9). GBD 2021 provides the latest comprehensive analysis of 371 diseases and 87 risk factors across 204 countries and regions worldwide [7]. In this study, we included the prevalence and DALY data for blindness and vision loss from 1990 to 2021 and conducted subgroup analyses by gender, age, specific causes, region, and country. Data were obtained using the Global Health Data Exchange (GHDx) query tool maintained by the Institute for Health Metrics and Evaluation (http://ghdx.healthdata.org/gbd-results-tool). This platform enables users to retrieve annual counts and age-standardised rates (ASRs) for disease prevalence, DALYs, and other health indicators, stratified by sex, age, region, and country [12]. Because blindness and vision impairment are considered non-fatal conditions in the GBD framework (years of life lost (YLL) = 0), the DALY estimates presented in this study are equivalent to years lived with disability (YLD), representing a severity-weighted measure of health loss [13]. By leveraging the GBD database on blindness and vision loss, this study systematically evaluates temporal trends and distribution patterns across different populations and geographic regions, providing important evidence to inform targeted prevention and control strategies for blindness and vision impairment across specific time periods and age groups worldwide. Our study utilised anonymised, publicly available epidemiological data, which did not require ethical approval or patient informed consent.

### Global descriptive estimation of blindness and vision loss

Descriptive analysis was used to assess the burden of blindness and vision loss at the global, regional, and national levels. Additionally, based on the SDI, which combines total fertility rate, mean education level, and per capita income, global regions were classified into low SDI (<0.466), low-middle SDI (0.466 ~ 0.619), middle SDI (0.619 ~ 0.712), high-middle SDI (0.712–0.810), and high SDI (≥ 0.810) [14,15]. In this study, we also utilised a world map of diseases to visualise the age-standardised prevalence rate (ASPR) and ASR of DALYs for blindness and vision loss across 204 countries. We performed Joinpoint analysis to evaluate the growth trends of blindness and vision loss caused by different causes worldwide. Furthermore, we provided descriptive analysis of blindness and vision loss in 21 regions within seven super regions globally, as well as for the five SDI-level regions. The study population included all age groups from 0 to 95 years and above, and we conducted age subgroup analyses in 5-year intervals. Spearman correlation analysis was also performed to assess the relationship between SDI levels and the ASPR and ASR of DALYs across different regions and countries globally in 2021.

### Projection analysis

The BAPC model was employed to project the prevalence, DALYs, and ASRs of blindness and vision loss from 2022 to 2050. This model specifies a Poisson likelihood with a log link function, utilising a Bayesian Age-Period-Cohort framework with second-order random walk (RW2) priors for age, period, and cohort effects to smooth temporal trends and enhance forecasting stability [16]. The RW2 structure was selected to capture nonlinear dynamics while penalising excessive complexity via Integrated Nested Laplace Approximations (INLA). Crucially, our projections incorporated GBD 2021 demographic forecasts, using age- and sex-specific population projections (2022–2050) as the denominator to account for future population growth and aging. To evaluate the impact of potential health interventions, we conducted scenario-based sensitivity analyses. In addition to the baseline projection, we simulated an 'optimistic scenario' (an annual 1% decline in age-specific rates) and a 'pessimistic scenario' (an annual 1% increase in age-specific rates) [17,18]. These scenarios represent a range of potential outcomes for global health strategic planning, independent of the probabilistic uncertainty captured by the model's 95% credible intervals (CrI). To validate the model’s predictive performance, we conducted back-testing by training the BAPC model on historical data from 1990–2010 to project trends for 2011–2021. The observed GBD estimates for 2021 were found to be consistent with the 95% CrIs of our back-casted projections, confirming the model’s reliability. Analysis was performed using the *R* package BAPC, version 0.0.36 (R statistical computing environment, R Core Team, Vienna, Austria) and INLA, version 23.09.09 (R Core Team, Vienna, Austria).

### Statistical analysis

The prevalence and DALY estimates reported in this study are presented with 95% uncertainty intervals (UIs) [19]. These intervals were defined as the 2.5th and 97.5th percentiles of the 1000 posterior draws from the GBD study [20]. While all downstream analyses-including Joinpoint regression, correlation analyses, and BAPC projections-were primarily conducted using the mean estimates provided by the GBD study, the reported 95% UIs were utilised to reflect the inherent variability of the modelled estimates. Although uncertainty from the full set of 1000 posterior draws was not propagated through every sequential step, the BAPC model inherently accounts for uncertainty through its Bayesian inference framework (INLA), providing 95% CrI for all 2050 projections [21]. To ensure comparability across different populations, regions, and time periods, ASRs were calculated based on the GBD 2021 standard population. Temporal trends in age-standardised prevalence and DALY rates were characterised using the Joinpoint Regression Program, version 4.9.0.0 (National Cancer Institute, Rockville, MD, USA). We employed a log-linear model to estimate the annual percent change (APC) and the average annual percent change (AAPC), along with their corresponding 95% CIs [22]. To prevent overfitting during the 1990–2021 study period, the maximum number of joinpoints was capped at 5. The Monte Carlo permutation test was utilised as the selection criterion to identify the most statistically significant inflection points (*P* < 0.05) [23]. To identify and rank regions and countries with the ‘fastest growth’ in disease burden, the magnitude of the AAPC was employed as the primary evaluative metric. This approach ensures that the identified trends reflect age-standardised epidemiological shifts across the 32-year study period, effectively isolating the underlying disease dynamics from fluctuations in population size or demographic aging [24,25]. Additionally, to account for the heteroscedasticity inherent in GBD estimates, each Joinpoint regression model was weighted by the inverse of the standard error of the rates. All statistical analyses were performed using *R*, version 4.3 (R Core Team, Vienna, Austria) and the Joinpoint Regression Program, version 4.9.0.0 (National Cancer Institute, Rockville, Maryland, USA). Key R packages included BAPC for Bayesian age-period-cohort forecasting, INLA (Version 23.09.09) for latent Gaussian inference, and ggplot2 (Version 3.4.2) for high-quality data visualisation. Detailed scripts for independent replication are available at our GitHub repository (URL: https://github.com/CurryevtWSN/Blindness-and-Vision-Loss-2021/tree/main). A two-sided *P*-value <0.05 was considered the threshold for statistical significance.

## RESULTS

### Global distribution of blindness and vision loss

According to the GBD 2021 framework, the global burden of blindness and vision loss-an aggregate category encompassing blindness, moderate-to-severe distance vision impairment [7,26], mild distance impairment, and presbyopia-related near vision loss-has increased significantly over the past three decades. The total prevalence rose from 547.02 million (95% UI = 451.54, 667.60 million) in 1990 to 1350.05 million (95% UI = 1087.46, 1673.98 million) in 2021. This represents approximately 17% of the global population in 2021, reflecting a broad spectrum of visual needs ranging from refractive correction to surgical intervention for blinding conditions. Correspondingly, the ASPR increased from 12 453.52 per 100 000 (95% UI = 10 287.58, 15 226.09) to 15 784.33 per 100 000 (95% UI = 12 761.44, 19 502.32), with an AAPC of 0.85 (95% CI = 0.7, 1.11, *P* < 0.001). On the other hand, globally, DALY increased from 14.3132 million (95% UI = 9.8122, 20.3425 million) in 1990 to 29.1640 million (95% UI = 19.0335, 42.9173 million) in 2021; the corresponding ASR of DALY changed from 342.01 (95% UI = 237.87, 482.17) in 1990 to 342.78 (95% UI = 224.21, 503.61) in 2021, with an AAPC of 0.05 (95% CI = −0.04, 0.2) (*P* = 0.298). Notably, among the causes of blindness and vision loss, near vision loss showed a significant increase from 1990 to 2021, with prevalence rising from 427.9377 million (95% UI = 322.7762, 561.6451 million) in 1990 to 1155.0630 million (95% UI = 875.2261, 1514.5975 million) in 2021, and the corresponding ASPR growing from 9788.66 (95% UI = 7371.64, 12 888.27) in 1990 to 13 436.18 (95% UI = 10 223.45, 17 585.84) in 2021, with an AAPC of 1.14 (95% CI = 0.94, 1.51) (*P* < 0.001). Other causes exhibited the following trends in prevalence from 1990 to 2021: age-related macular degeneration with an AAPC of −0.21 (95% CI = −0.3, −0.15, *P* < 0.001), cataract with an AAPC of 0.25 (95% CI = 0.16, 0.33, *P* < 0.001), glaucoma with an AAPC of −0.74 (95% CI = −0.81, −0.66, *P* < 0.001), refraction disorders with an AAPC of −0.2 (95% CI = −0.28, −0.16, *P* < 0.001), and other vision loss with an AAPC of −0.39 (95% CI = −0.44, −0.35, *P* < 0.001) (**Table 1**).

**Table 1:** Changes in prevalence and DALY for blindness and vision loss caused by different conditions globally from 1990 to 2021

Measure	Causes	1990		2021		ASR AAPC	*P*-value
		**Number (95% UI)**	**Rate (95% UI)**	**Number (95% UI)**	**Rate (95% UI)**		
**Prevalence**							
	Blindness and vision loss	547 017 796.7 (451 543 289.53, 667 598 560.32)	12 453.52 (10 287.58, 15 226.09)	1 350 054 705.21 (1 087 458 891.73, 1 673 977 885.6)	15 784.33 (12 761.44, 19 502.32)	0.85 (0.7, 1.11)	<0.001
	Age-related macular degeneration	3 640 179.66 (3 037 098.06, 4 353 902.22)	99.5 (83.16, 118.04)	8 057 520.62 (6 705 284.35, 9 823 237.6)	94.00 (78.32, 114.42)	−0.21 (−0.3, −0.15)	<0.001
	Cataract	42 331 734.62 (37 402 759.62, 47 527 331.71)	1145.05 (1022.69, 1278.12)	100 570 685.81 (88 771 984.68, 114 032 510.52)	1181.01 (1044.47, 1334.29)	0.25 (0.16, 0.33)	<0.001
	Glaucoma	4 072 106.53 (3 489 888.71, 4 752 867.32)	116.29 (100.81, 136.3)	7 587 672.87 (6 522 905.98, 8 917 725.43)	90.12 (77.78, 105.49)	−0.74 (−0.81, −0.66)	<0.001
	Near vision loss	427 937 731.06 (322 776 201.96, 561 645 080.04)	9788.66 (7371.64, 12 888.27)	1 155 063 049.59 (875 226 145.9, 1 514 597 489.37)	13 436.18 (10 223.45, 17 585.84)	1.14 (0.94, 1.51)	<0.001
	Refraction disorders	95 978 319.48 (86 236 196.7, 106 044 403.07)	2053.56 (1835.31, 2275.8)	159 765 916.65 (142 526 915.12, 178 698 347.92)	1919.66 (1715.24, 2135.28)	−0.2 (−0.28, −0.16)	<0.001
	Other vision loss	20 644 292.72 (18 414 360.31, 23 016 151.55)	507.89 (452.94, 568.05)	38 767 272.25 (34 102 057.4, 43 775 623.66)	454.75 (402.01, 511.73)	−0.39 (−0.44, −0.35)	<0.001
**DALYs**	Blindness and vision loss	14 313 207.69 (9 812 220.76, 20 342 527.35)	342.01 (237.87, 482.17)	29 163 992.74 (19 033 505.67, 42 917 325.83)	342.78 (224.21, 503.61)	0.05 (−0.04, 0.2)	0.298
	Age-related macular degeneration	302 902.28 (206 475.32, 421 951.89)	8.38 (5.7, 11.53)	578 020.38 (401 241.32, 797 569.82)	6.78 (4.7, 9.32)	−0.81 (−0.92, −0.72)	<0.001
	Cataract	3 416 560.49 (2 475 133.38, 4 545 239.37)	91.06 (66.26, 120.4)	6 553 846.48 (4 736 091.92, 8 804 276.71)	76.97 (55.64, 103.5)	−0.44 (−0.5, −0.39)	<0.001
	Glaucoma	467 600.4 (323 490.51, 648 641.64)	13.37 (9.36, 18.5)	759 900.23 (530 942.9, 1 049 127.17)	9.05 (6.29, 12.46)	−1.21 (−1.32, −1.12)	<0.001
	Near vision loss	4 316 104.57 (1 937 499.87, 8 410 596.3)	98.26 (44.11, 190.99)	11 649 944.75 (5 214 018.65, 22 421 658.63)	135.52 (60.82, 260.39)	1.16 (0.96, 1.53)	<0.001
	Refraction disorders	4 029 083.94 (2 821 217.2, 5 812 173.79)	88.04 (62.19, 125.15)	6 618 600.07 (4 599 082.32, 9 528 675.98)	79.11 (54.94, 114.14)	−0.33 (−0.37, −0.3)	<0.001
	Other vision loss	1 780 956 (1 256 667.81, 2 420 455.2)	42.89 (30.57, 58.13)	3 003 680.83 (2 148 641.6, 4 068 865.01)	35.35 (25.38, 47.88)	−0.66 (−0.72, −0.6)	<0.001

ASR – age-standardised rate, AAPC – average annual percent change, DALYs – disability-adjusted life years, UI – uncertainty interval

### The distribution of blindness and vision loss at regional and national levels

**Figure 1:**
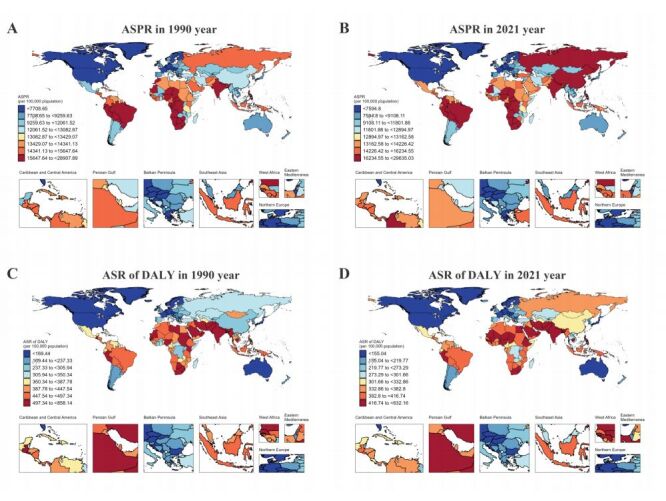
Global distribution maps of ASPR and ASR of DALY for blindness and vision loss in 1990 and 2021. **Panel A**. The global map of ASPR for blindness and vision loss in 1990. **Panel B**. The global map of ASPR for blindness and vision loss in 2021. **Panel C.** The global map of ASR of DALY for blindness and vision loss in 1990. **Panel D.** The global map of ASR of DALY for blindness and vision loss in 2021. ASPR – age-standardised prevalence rate, ASR – age-standardised rate, DALY – disability adjusted life year. Data derived from the GBD database and the world map base layer used for visualisation were obtained from the China Geographic Information Resource and Network.

We divided the global regions based on seven different GBD super regions and five SDI regions. The results show that the highest prevalence occurred in South Asia and East Asia. In South Asia, the prevalence increased from 128.1250 million (95% UI = 106.0099, 156.6590 million) in 1990 to 402.1270 million (95% UI = 319.6165, 496.5042 million) in 2021; with an AAPC of 1.15 (95% CI = 0.84, 1.67, *P* < 0.001) for ASPR. In East Asia, the prevalence rose from 118.3248 million (95% UI = 94.6142, 148.0156 million) in 1990 to 366.3642 million (95% UI = 281.8202, 472.9756 million) in 2021, with an AAPC of 1.18 (95% CI = 0.96, 1.53, *P* < 0.001) for ASPR. At the SDI region level, the prevalence in the middle SDI region increased from 174.4760 million (95% UI = 143.8424, 213.5471 million) in 1990 to 495.4009 million (95% UI = 395.9792, 617.9326 million) in 2021, with an AAPC of 0.89 (95% CI = 0.71, 1.2, *P* < 0.001 for ASPR (**Table 2**). Among the 204 countries globally, the fastest-growing countries in terms of prevalence of blindness and vision loss are India, China, and Nepal. In India, the ASPR increased from 19 125.79 (95% UI = 16 070.44, 22 994.75) in 1990 to 26 333.59 (95% UI = 21 116.25, 32 187.03) in 2021, with an AAPC of 1.35 (95% CI = 1.01, 1.9, *P* < 0.001. In China, the ASPR increased from 12 068.81 (95% UI = 9799.7, 14 928.08) in 1990 to 17 428.86 (95% UI = 13 625.18 to 22 177.12) in 2021, with an AAPC of 1.23 (95% CI = 0.99, 1.59, *P* < 0.001). In Nepal, the ASPR increased from 20 515.7 (95% UI = 16 427.15, 25 498.41) in 1990 to 25 663.58 (95% UI = 21 476.47, 29 336.61) in 2021, with an AAPC of 0.91 (95% CI = 0.51, 1.64, *P* < 0.001). On the other hand, in terms of DALY, the fastest-growing countries are Côte d'Ivoire, Burkina Faso, and Benin. In Côte d'Ivoire, the ASR increased from 283.46 (95% UI = 184.1, 433.74) in 1990 to 408.76 (95% UI = 282.28, 584.18) in 2021, with an AAPC of 1.19 (95% CI = 1.08, 1.3, *P* < 0.001). In Burkina Faso, the ASR increased from 259.89 (95% UI = 183.4, 361.83) in 1990 to 341.71 (95% UI = 245.32, 472.56) in 2021, with an AAPC of 0.82 (95% CI = 0.73, 0.93, *P* < 0.001). In Benin, the ASR increased from 358.08 (95% UI = 245.95, 508.74) in 1990 to 433.73 (95% UI = 305.82, 612.13) in 2021, with an AAPC of 0.59 (95% CI = 0.55, 0.65, *P* < 0.001) (Table S2 and S3 in the **Online Supplementary Document**). **Figure 1** also shows the global maps of ASPR and ASR of DALY for 1990 and 2021. The AAPC values of ASPR and ASR of DALY for 204 countries are presented in Figure S1 in the **Online Supplementary Document****.**

**Table 2:** Trends in blindness and vision loss globally and across 26 regions from 1990 to 2021

Measure	Location	1990 y		2021 y		ASR AAPC	*P*-value
		**Number (95%UI)**	**Rate (95%UI)**	**Number (95%UI)**	**Rate (95%UI)**		
**Prevalence**							
	Global	547 017 796.7 (451 543 289.53, 667 598 560.32)	12 453.52 (10 287.58, 15 226.09)	1 350 054 705.21 (1 087 458 891.73, 1 673 977 885.6)	15 784.33 (12 761.44, 19 502.32)	0.85 (0.7, 1.11)	<0.001
	Central Europe, Eastern Europe, and Central Asia						
	*Central Asia*	5 999 320.65 (50 93 133.83, 7 058 559.86)	11 678.57 (9877.35, 13 919.76)	9 800 231.99 (8 063 714.27, 11 958 047.22)	11 337.99 (9395.86, 13 709.18)	−0.1 (−0.12, −0.07)	<0.001
	*Central Europe*	11 301 803.2 (9 163 590.72, 14 068 610.29)	7927.47 (6448.7, 9814.26)	14 656 019.21 (11 674 712, 18 582 295.94)	7815.1 (6335.36, 9705.11)	−0.02 (−0.03, 0.00)	0.044
	*Eastern Europe*	36 734 559.89 (29 713 560.93, 45 423 801.24)	13 779.57 (11 253.96, 16 853.33)	48 983 955.78 (38 948 518.38, 60 747 065.67)	16 024.9 (12 870.73, 19 877.3)	0.69 (0.52, 0.87)	<0.001
	High-income						
	*Australasia*	1 795 167.46 (1 413 869.2, 2 290 554.77)	8190.31 (6499.87, 10 545.61)	3 401 226.92 (2 681 729.4, 4 292 602.55)	8133.7 (6464.58, 10396.57)	−0.02 (−0.03, −0.01)	<0.001
	*High-income Asia Pacific*	16 789 211.58 (13 002 681.81, 21 997 110.05)	8425.87 (6603.56, 10 858.65)	25 894 854.23 (20 355 821.58, 32 867 818.71)	8264.38 (6506.89, 10 712.39)	−0.07 (−0.08, −0.05)	<0.001
	*High-income North America*	20 971 763.83 (16 558 226.37, 26 563 163.1)	6753.33 (5315.23, 8661.61)	36 446 153.84 (28 221 491.44, 46 795 779.14)	7254.73 (5572.89, 9567.56)	0.78 (0.54, 1.01)	<0.001
	*Southern Latin America*	4 396 056.78 (3 601 622.32, 5 474 477.23)	9326.08 (7648.84, 11 690.08)	7 262 775.17 (5 885 038.82, 9 134 258.26)	9155.05 (7433.43, 11 493.15)	−0.05 (−0.06, −0.04)	<0.001
	*Western Europe*	40 595 387.93 (32 761 286.55, 50 940 857.49)	8121.61 (6585.85, 10 082.19)	56 165 678.34 (44 715 731.95, 71 065 738.3)	7946.29 (6454.99, 9908.51)	−0.06 (−0.07, −0.05)	0.002
	Latin America and Caribbean						
	*Andean Latin America*	4 318 984.52 (3 532 166.33, 5 306 747.26)	16 903.63 (13 882.65, 20 951.08)	10 356 203.84 (8 361 492.76, 12 918 661.84)	16 476.21 (13 371.19, 20 383.15)	−0.06 (−0.07, −0.05)	<0.001
	*Caribbean*	3 979 414.74 (3 260 766.36, 4 885 423.68)	13 755.73 (11 213.2, 16 968.62)	7 038 548.59 (5 657 272.35, 8 794 606.06)	13485 (10 863.14, 16 757.52)	−0.03 (−0.05, −0.01)	0.002
	*Central Latin America*	14 764 942.61 (12 561 411.91, 17 429 730.4)	13 411.51 (11 405.09, 15 696.34)	37 315 389.12 (30 257 428.65, 46 381 473.73)	14 453.02 (11 764.79, 17 836.47)	0.53 (0.37, 0.67)	<0.001
	*Tropical Latin America*	20 143 760.76 (16382337.83, 25 230 456.08)	17 482.3 (14 238.68, 21 641.88)	43 813 494.4 (35 238 392.76, 55 038 651.43)	17 212.58 (14 001.93, 21 361.7)	−0.01 (−0.14, 0.12)	<0.001
	North Africa and Middle East	29 263 787.93 (25 105 975.25, 33 767 560.73)	13 453.53 (11 500.35, 15 582.05)	72 705 926.2 (59 552 709.82, 90 451 833.69)	13 748.25 (11 466.94, 16 736.27)	0.09 (0.06, 0.13)	<0.001
	South Asia	128 124 984.17 (106 009 920.55, 156 659 019.38)	18 295.63 (15 416.1, 22 033.96)	402 126 985.85 (319 616 472.42, 496 504 212.92)	24 004.88 (19 437.79, 29 178.14)	1.15 (0.84, 1.67)	<0.001
	Southeast Asia, East Asia, and Oceania						
	*East Asia*	118 324 796.52 (94 614 188.63, 148 015 602.04)	12 122.95 (9829.17, 15 053.58)	366 364 186.61 (281 820 231.43, 472 975 587.67)	17 290.19 (13 510.09, 22 013.92)	1.18 (0.96, 1.53)	<0.001
	*Oceania*	529 959.65 (435 370.49, 655 133.4)	14 057.75 (11 711.82, 17 063)	1 295 971.14 (1 059 371.27, 1 622 888.72)	13 901.06 (11 549.69, 16 951.4)	−0.07 (−0.09, −0.06)	<0.001
	*Southeast Asia*	41 869 342.52 (34 898 188.1, 50 889 897.96)	13 393.94 (11 297.93, 15 980.17)	94 066 377.96 (76 452 801.26, 116 543 788.23)	13 534.38 (11 239.4, 16 425.14)	0.07 (0.04, 0.1)	<0.001
	Sub-Saharan Africa						
	*Central sub-Saharan Africa*	6 490 085.69 (4 949 980.55, 8 524 700.84)	19 643.15 (15 427.08, 25 230.16)	16 861 024.95 (12 830 614.55, 22 501 510.49)	19 529.79 (15 323.8, 24 996.06)	−0.02 (−0.02, 0)	0.049
	*Eastern sub-Saharan Africa*	13 253 286.54 (10 694 966.77, 16 552 849.71)	12 991.75 (10 629.82, 15 877.08)	31 264 385.46 (24 981 569.33, 39 666 568.23)	12 933.19 (10 538.65, 15 826.76)	0.02 (−0.02, 0.06)	0.428
	*Southern sub-Saharan Africa*	9 493 073.66 (7 433 496.26, 12 252 411.19)	25 919.64 (20 632.61, 32 508.56)	19 167 216.73 (14 971 876.63, 24 303 394.04)	26 595.25 (21 441.32, 32 958.12)	0.34 (0.11, 0.77)	0.735
	*Western sub-Saharan Africa*	17 878 106.05 (14 269 995.69, 22 773 608.19)	15 549.07 (12 581.82, 19 596.09)	45 068 098.89 (36 160 263.23, 57 854 860.21)	15 805.12 (12 881.45, 19 632.85)	0.11 (0.08, 0.15)	<0.001
	SDI region						
	*Low SDI*	46 351 233.92 (37 973 176.98, 57 917 646.88)	15 929.08 (13 171.45, 19 413.3)	122 776 976.4 (99 375 997.64, 153 251 122.8)	17 906.62 (14 739.72, 21 711.32)	0.49 (0.35, 0.75)	<0.001
	*Low-middle SDI*	124 621 695.02 (104 153 149.77, 150 755 086.74)	16 736.87 (14 129.17, 20 229.92)	319 482 912.81 (259 780 277.27, 393 412 368.98)	19 463.65 (16 021.28, 23 577.39)	0.64 (0.43, 1.01)	<0.001
	*Middle SDI*	174 475 958.72 (143 842 418.72, 213 547 138.23)	14 054.59 (11 660.67, 16 988.55)	495 400 856.23 (395 979 183.72, 617 932 624.64)	17 934.58 (14 509.78, 22 121.77)	0.89 (0.71, 1.2)	<0.001
	*High-middle SDI*	119 243 109.89 (96 869 096.83, 146 669 025.22)	11 633.25 (9530.55, 14 208.78)	271 632 316.21 (214 672 806.55, 341 463 775.9)	14 902.03 (11 921.27, 18 650.37)	0.83 (0.71, 0.97)	<0.001
	*High SDI*	81 855 813.29 (64 910 208.15, 102 923 672.98)	8077.8 (6470.39, 10 184.36)	139 974 758.34 (111 049 479.62, 176 055 510.25)	8638.17 (6887, 10 881.74)	0.29 (0.21, 0.41)	<0.001
**DALYs**	Global	14 313 207.69 (9 812 220.76, 20 342 527.35)	342.01 (237.87, 482.17)	29 163 992.74 (19 033 505.67, 42 917 325.83)	342.78 (224.21, 503.61)	0.05 (−0.04, 0.2)	0.298
	Central Europe, Eastern Europe, and Central Asia						
	*Central Asia*	161 348.46 (111 039.84, 230 609.93)	328.82 (228.87, 472.45)	241 938.61 (162 849.97, 350 818.93)	294.15 (201.84, 427.41)	−0.35 (−0.36, −0.33)	<0.001
	*Central Europe*	24 1091.98 (156 719.41, 361 407.76)	171.24 (111.87, 254.63)	305 426.13 (199 910.67, 464 972.81)	159.17 (101.94, 240.13)	−0.23 (−0.23, −0.22)	<0.001
	*Eastern Europe*	816 644.95 (535 182.42, 1 212 199.99)	310.86 (204.43, 458.72)	977 038.14 (614 682.24, 1 505 907.66)	312.36 (194.32, 483)	0.08 (−0.01, 0.18)	0.08
	High-income						
	*Australasia*	35 564.15 (22 437.81, 53 336.12)	162.9 (102.74, 243.55)	66 933.29 (42 492.26, 100 671.3)	155.97 (96.85, 237.29)	−0.12 (−0.15, −0.1)	<0.001
	*High-income Asia Pacific*	322 625.91 (203 932.46, 487 617.19)	167 (106.14, 249.49)	520 737.37 (343 506.06, 782 392)	155.97 (97.08, 238.95)	−0.23 (−0.25, −0.22)	<0.001
	*High-income North America*	423 646.89 (272 779.68, 629 632.77)	133.17 (84.24, 202.17)	693 288.16 (443 585.73, 1 047 807.6)	134.11 (83.8, 206.86)	0.14 (−0.01, 0.41)	0.068
	*Southern Latin America*	102 461.69 (67 362.26, 149 734.14)	220.17 (145.61, 320.1)	158 815.54 (102 761.59, 233 988.36)	198.25 (127.42, 292.54)	−0.32 (−0.35, −0.29)	<0.001
	*Western Europe*	965 422.19 (65 9431.12, 1 412 895.52)	191.28 (128.71, 278.3)	1 288 986.24 (880 809.86, 1 871 786.34)	174.14 (114.36, 257.08)	−0.3 (−0.3, −0.29)	<0.001
	Latin America and Caribbean						
	*Andean Latin America*	116 050.53 (79 303.1, 166 323.05)	494.54 (340.78, 704.04)	245 204.45 (163 103.91, 355 847.69)	400.19 (269.29, 580.72)	−0.72 (−0.74, −0.69)	<0.001
	*Caribbean*	96 469.88 (65 411.88, 139 043.53)	346.01 (237.45, 497.86)	156 476.95 (103 914.7, 228 983.47)	298.15 (197.79, 437.74)	−0.46 (−0.47, −0.45)	<0.001
	*Central Latin America*	396 259.02 (273 028.61, 560 374.36)	400.32 (282.1, 561.62)	868 110.64 (576 508.92, 1271263.21)	343.83 (230.44, 503.65)	−0.36 (−0.42, −0.3)	<0.001
	*Tropical Latin America*	503 024.08 (339 376.48, 720 530.04)	474.38 (328.07, 673.82)	1 015 658.64 (675 894.04, 1 480 786.08)	405.41 (271.52, 592.24)	−0.42(−0.52, −0.32)	<0.001
	North Africa and Middle East	907 508.52 (635 252.11, 1 252 872.82)	471.16 (338.64, 647.39)	1 878 964.29 (1 275 378.9, 2 722 790.62)	386.78 (270.54, 545.75)	−0.69 (−0.73, −0.65)	<0.001
	South Asia	4 246 415.53 (2 998 688.11, 5 910 274.77)	705.16 (506.27, 962.9)	9 347 184.52 (6 144 744.64, 13 547 486.99)	605.29 (407, 862.35)	−0.44 (−0.56, −0.27)	<0.001
	Southeast Asia, East Asia, and Oceania						
	*East Asia*	2 630 286.99 (1 758 777.34, 3 854 854.25)	293.43 (201.14, 427.4)	6 635 112.8 (4 118 479.22, 10 490 707.25)	318.92 (200.39, 497.18)	0.47 (0.27, 0.67)	0.003
	*Oceania*	13 737.83 (9325.95, 19 719.32)	419.15 (294, 593.77)	31 034.19 (20 774.81, 45 340.82)	374.47 (256.98, 538.13)	−0.44 (−0.49, −0.41)	<0.001
	*Southeast Asia*	1 236 638.06 (860 337.01, 1 737 083.96)	453.08 (321.23, 626.13)	2 346 142.54 (1 591 101.07, 3 405 372.61)	361.6 (252.27, 517.68)	−0.76 (−0.78, −0.73)	<0.001
	Sub-Saharan Africa						
	*Central sub-Saharan Africa*	97 360.21 (57 542.07, 163 105.72)	335.65 (205.97, 532.41)	246 097.4 (142 562.33, 413 357.26)	324.03 (195.47, 518.98)	−0.11(−0.12, −0.10)	<0.001
	*Eastern sub-Saharan Africa*	360 862.74 (249 515.66, 499 645.56)	421.46 (301.34, 580.71)	759 624.95 (516 289.9, 1 082 795.98)	374.77 (263.77, 518.96)	−0.33 (−0.37, −0.29)	<0.001
	*Southern sub-Saharan Africa*	171 352.94 (107 980.39, 264 204.31)	523.31 (344.11, 781.52)	312 083.38 (190 168.91, 497 267.77)	473.88 (299.35, 723)	−0.23 (−0.34, 0.00)	0.05
	*Western sub-Saharan Africa*	468 435.15 (321 868.81, 662 594.66)	474.83 (337.31, 660.5)	1 069 134.51 (719 353.56, 1 540 309.82)	449.82 (315.5, 631.6)	−0.17 (0.02, −0.14)	<0.001
	SDI region						
	*Low SDI*	1 290 767.59 (891 197.75, 1 806 216.39)	524.07 (374.66, 719.54)	2 786 361.81 (1 848 668.2, 4 029 390.4)	473.57 (326.3, 660.44)	−0.24 (−0.29, −0.19)	<0.001
	*Low-middle SDI*	3 945 746.71 (2 769 679.14, 5 477 179.78)	606.86 (434.81, 833.54)	7 697 679.2 (5 131 661.75, 11 021 844.29)	509.85 (347.87, 719.48)	−0.53 (−0.61, −0.4)	<0.001
	*Middle SDI*	4 591 249.25 (3 148 132.21, 6 492 763.43)	413.76 (289.32, 580.14)	10 554 043.19 (6 846 917.15, 15 616 729.53)	396.12 (260.36, 577.94)	0.00 (−0.16, 0.15)	0.993
	*High-middle SDI*	2 785 702.47 (1 871 708.31, 4 042 205.93)	282.18 (193.2, 408.21)	5 362 632.8 (3 413 656.15, 8 216 308.01)	293.72 (186.7, 449.95)	0.17 (0.06, 0.34)	0.004
	*High SDI*	1 688 376.41 (109 6641.29, 2510 669.48)	166.06 (107.07, 246.44)	2 745 288.24 (1 774 400.86, 4 162 106.09)	164.31 (102.75, 249.44)	−0.02 (−0.07, 0.05)	0.399

ASR – age-standardised rate, AAPC – average annual percent change, DALYs – disability-adjusted life years, UI – uncertainty interval

### Trends analysis results for blindness and vision loss by gender and disease subgroups

**Figure 2:**
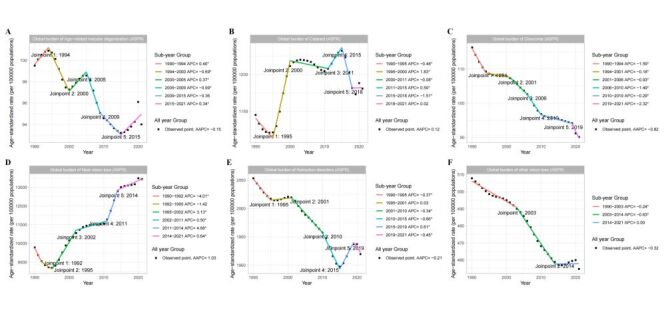
Trend analysis of ASPR for blindness and vision loss caused by different causes from 1990 to 2021. **Panels A–F** correspond to the ASPR trend results for blindness and vision loss caused by age-related macular degeneration, cataract, glaucoma, near vision loss, refraction disorders, and other vision loss, respectively. ASPR – age-standardised prevalence rate, AAPC – average annual percent change, APC – annual percent change.

By evaluating the trends of blindness and vision loss from 1990 to 2021 by gender subgroups, the results show that females have significantly higher ASPR and ASR of DALY for blindness and vision loss compared to males. When considering both genders, the growth trend in ASPR for blindness and vision loss slowed between 2014 and 2021 compared to 2011 to 2014 (APC = 0.49 *vs*. 3.61). Additionally, for ASR of DALY, blindness and vision loss showed a significant increase from 2011 to 2014, with an APC of 1.24, while a declining trend occurred from 2014 to 2021, with an APC of −0.12 (Figure S2 in the **Online Supplementary Document**). Moreover, the trend analysis of blindness and vision loss caused by different conditions from 1990 to 2021 reveals the following results for ASPR: blindness and vision loss caused by age-related macular degeneration had an AAPC of −0.15 from 1990 to 2021 but showed a partial upward trend from 2015 to 2021, with an APC of 0.34. Blindness and vision loss caused by cataracts had an AAPC of 0.12 from 1990 to 2021, with the fastest growth period from 1995 to 2000, where the APC was 1.83. Blindness and vision loss caused by glaucoma had an AAPC of −0.82 from 1990 to 2021, with the fastest decline from 2019 to 2021, where the APC was −2.32. Blindness and vision loss caused by near vision loss had an AAPC of 1.03 from 1990 to 2021, with the fastest growth period from 2011 to 2014, where the APC was 4.68. Blindness and vision loss caused by refraction disorders had an AAPC of −0.21 from 1990 to 2021, with the fastest decline from 2010 to 2015, where the APC was −0.66. Blindness and vision loss caused by other vision loss had an AAPC of −0.32 from 1990 to 2021, with the fastest decline from 2003 to 2014, where the APC was −0.63 (**Figure 2**). Additionally, we have also provided the trends in ASR of DALY caused by different causes leading to blindness and vision loss from 1990 to 2021 (Figure S3 in the **Online Supplementary Document**).

### The correlation between blindness and vision loss and SDI levels

**Figure 3:**
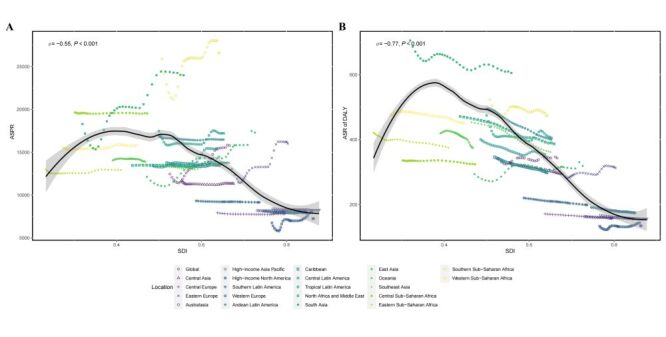
Correlation analysis results between ASPR and ASR of DALY for blindness and vision loss and SDI levels globally and across 21 regions from 1990 to 2021. **Panel A.** The correlation analysis between ASPR and SDI levels globally and across 21 regions. **Panel B.** The correlation analysis between ASR of DALY and SDI levels globally and across 21 regions. ASPR – age-standardised prevalence rate, ASR – age-standardised rate, DALY – disability adjusted life year, SDI – sociodemographic index.

We conducted a Spearman correlation analysis between SDI levels and ASPR and ASR of DALY at both global and 21 region levels. The results showed a significant negative correlation between ASPR and SDI levels globally and across the 21 regions (Spearman’s rank correlation coefficient, ρ = −0.55, *P* < 0.001), as well as a significant negative correlation between ASR of DALY and SDI levels globally and across the 21 regions (Spearman’s rank correlation coefficient, ρ = −0.77, *P* < 0.001) (**Figure 3**). Additionally, we performed a Spearman correlation analysis between SDI levels and ASPR and ASR of DALY in 204 countries globally in 2021. The results indicated a significant negative correlation between ASPR and SDI in 2021 (Spearman’s rank correlation coefficient, ρ = −0.62, *P* < 0.001), and a significant negative correlation between ASR of DALY and SDI levels (Spearman’s rank correlation coefficient, ρ = −0.72, *P* < 0.001) (Figure S4 in the **Online Supplementary Document**). Additionally, for the global level and five SDI regions, we evaluated the differences in ASPR and ASR of DALY for blindness and vision loss, as well as its six specific causes, across these regions. The results showed that blindness and vision loss had the highest ASPR and ASR of DALY in the low-middle SDI region. Among its specific causes, for the ASPR metric, age-related macular degeneration and glaucoma were primarily concentrated in the low SDI region, while the other four causes were mainly concentrated in the low-middle SDI region. For the ASR of DALY metric, age-related macular degeneration, glaucoma, and other vision loss were mainly concentrated in the low SDI region, whereas the other three causes were predominantly concentrated in the low-middle SDI region (Figure S5 in the **Online Supplementary Document**).

### Subgroup analysis of the causes of blindness and vision loss

We conducted a cause analysis of blindness and vision loss across age subgroups, ranging from zero to over 95 years old. The results showed that for the prevalence metric, the leading cause of blindness and vision loss was near vision loss, which exhibited a trend of increasing with age before declining, with the highest proportion observed in the 45 to 49-year-old group. In females, near vision loss accounted for 90.3%, while in males, it accounted for 89.9%. Refraction disorders showed a trend of gradually decreasing with age before slightly increasing, with the highest proportion observed in the <5-year-old age group, accounting for 56.0% in females and 60.2% in males. For the DALY metric, the most significant change was seen in blindness and vision loss caused by cataracts, with the proportion increasing with age, peaking in the 85 to 89-year-old group. Cataracts accounted for 48.6% in females and 46.5% in males (Figure S6 in the **Online Supplementary Document**). We also included a comparison of the prevalence and DALY numbers and rates for blindness and vision loss across different age groups in 1990 and 2021. The results showed that in terms of prevalence, the age group with the highest number of cases was 55–59 years in 1990 and 50–54 years in 2021. For DALY numbers, the age group with the highest DALYs in both 1990 and 2021 was 65–69 years. Regarding prevalence and DALY rates, both showed a significant increase with age in 1990 and 2021 (Figure S7 in the **Online Supplementary Document**). Lastly, we supplemented the analysis with data on the prevalence number, ASPR, DALY number, and ASR of DALY for blindness and vision loss and its six causes across the globe and in 26 regions from 1990 to 2021. Regarding the numerical expansion of the burden, South Asia exhibited the most rapid increase in the total number of individuals affected by blindness and vision loss, with a 213.9% rise from 1990 to 2021. Cause-specific analyses revealed divergent patterns of numerical growth across regions: cataracts increased most significantly in East Asia (247.2%), while near vision loss showed the sharpest elevation in South Asia (272.2%). Other notable regional increases in prevalence counts included age-related macular degeneration in Tropical Latin America (202.9%), refraction disorders in Western sub-Saharan Africa (171.9%), glaucoma in high-income Asia Pacific (160.2%), and other vision loss in Central sub-Saharan Africa (154.2%). While these figures primarily reflect the combined effects of population growth and demographic aging, they underscore the rapidly escalating demand for eye-care infrastructure in these specific regions. Detailed regional rankings and their corresponding percentage changes are available in Figure S8 in the **Online Supplementary Document**. Further changes in ASPR, DALY number, and ASR of DALY for blindness and vision loss and its six causes across the globe and 26 regions from 1990 to 2021 can be found in Figures S9–11 in the **Online Supplementary Document**.

### Blindness and vision loss BAPC prediction results

We used the BAPC model with a Poisson likelihood and RW2 priors to project the global burden of blindness and vision loss by gender subgroup through 2050. To ensure the reliability of these long-term projections, we first performed a formal back-testing validation by fitting the model with historical data from 1990–2010 to project trends for the 2011–2021 period. The results demonstrated that the observed GBD 2021 estimates for both prevalence and DALYs remained consistently within the 95% CrIs of our back-casted projections, confirming the robust predictive performance of our framework (Figure S12 in the **Online Supplementary Document**). The primary projection results show that, in the prevalence metric, the total prevalence is projected to reach 3144.2149 million by 2050, with a corresponding ASPR of 22 796.63. In the DALY metric, the total DALY is predicted to reach 57.7269 million by 2050, with a corresponding ASR of DALY of 392.12 (Figure S13 in the **Online Supplementary Document**). Additionally, we also conducted gender-based predictions of specific causes of blindness and vision loss up to 2050 (Figure S14 and Table S4 in the **Online Supplementary Document**).

## DISCUSSION

This study provides an updated and extended assessment of the global burden of blindness and vision loss through 2021, offering critical insights into the evolving epidemiological landscape in the post-COVID-19 era. By integrating granular stratifications across sex, age, and SDI levels, our analysis elucidates the complex distribution patterns and cause-specific dynamics of vision impairment worldwide. To address the inherent clinical and functional heterogeneity of the ‘blindness and vision loss’ category-which spans from presbyopia to total blindness-we intentionally utilised a dual-metric approach emphasising both prevalence and DALYs. While prevalence characterises the overall scale of health-system demand, DALYs (equivalent to YLDs in this non-fatal context) provide a severity-weighted measure of actual health loss. This strategic framework, further supported by the decomposition of the six primary aetiologies, ensures that the disproportionately high prevalence of milder conditions does not obscure the significant functional burden of sight-threatening diseases. Collectively, these findings provide a robust evidence base for tailoring targeted prevention strategies and optimising resource allocation across diverse regional and demographic contexts through 2050.

Globally, our study indicates that in 2021, 1350.0547 million people were affected by blindness and vision loss, with a corresponding ASPR of 15 784.33. Additionally, the DALY in 2021 reached 29.1640 million, with a corresponding ASR of DALY of 342.78. The ASPR of blindness and vision loss peaked in 2021. According to the World Bank database, the global population in 2021 was 7 888 408 686 [27,28], corresponding to an overall prevalence of vision impairment of approximately 17.11%, encompassing conditions ranging from near vision loss to total blindness. As early as 1999, the WHO, in collaboration with the International Agency for the Prevention of Blindness, jointly advocated for the global initiative to eliminate avoidable blindness (Vision 2020: The Right to Sight), aiming to eliminate 80% of avoidable blindness globally by 2020 [29]. However, current results indicate that this goal remains challenging. The initial five-year plan of the initiative primarily focused on controlling diseases causing blindness and vision loss, including cataracts, trachoma, onchocerciasis, childhood blindness, and uncorrected refractive disorders, among others. Our study also indicates that among the six different causes of blindness and vision loss globally, the ASPR for four diseases, including refraction disorders, age-related macular degeneration, glaucoma, and other vision loss, significantly declined from 1990 to 2021. However, the ASPR for blindness and vision loss caused by near vision loss and cataract significantly increased over the same period. The gradual rise in the prevalence of near vision loss and cataract may be related to the noticeable aging trends in certain countries and regions [30–32]. Furthermore, approximately 75% of the world's blind population is aged 50 years or older [33], and as early as 2018, the number of people aged 65 or older surpassed the number of children under five years for the first time in human history. Global population aging is expected to have a severe negative impact on age-related diseases, including cataract and age-related blindness caused by uncorrected near vision loss [29]. Thus, we must recognise that significant progress has been made in the global disease burden prevention and control of refraction disorders, age-related macular degeneration, glaucoma, and other vision loss. While continuing to enhance previous efforts, future priorities for disease prevention and control should focus on addressing blindness and vision loss caused by near vision loss and cataract.

The observed increase in global prevalence alongside relatively stable ASDRs warrants careful epidemiological interpretation. Several factors may explain this apparent divergence. First, global population ageing has substantially increased the number of individuals at risk of vision impairment, particularly for age-related conditions such as cataract [8,34,35]. Second, improvements in survival and health care have resulted in more individuals living longer with visual impairment, thereby increasing prevalence without proportionally increasing disability burden [36]. Third, enhanced detection and evolving diagnostic thresholds may have contributed to the identification of milder cases. Finally, changes in case definitions within successive iterations of the Global Burden of Disease Study, particularly the expanded inclusion of near vision loss, may have further increased prevalence estimates while exerting a more limited impact on DALY rates [7,37]. Taken together, these findings suggest that increasing prevalence does not necessarily indicate worsening disease severity, but rather reflects shifts in demographic structure, disease recognition, and methodological frameworks.

Among the 26 regions globally, we found that South Asia and East Asia were the regions with the fastest-growing prevalence of blindness and vision loss, while the middle SDI region was the SDI category with the fastest growth in ASPR. Referring to the world map analysis, we observed that the countries with the fastest-growing prevalence in South Asia and East Asia were mainly concentrated in India, China, and Nepal. In terms of ASPR, the primary cause of blindness and vision loss in East Asia was near vision loss. The GBD 2019 study on near vision loss in China also showed that the burden of near vision loss in China was most similar to that of the high-middle SDI region and exhibited significant differences from seven neighbouring countries, including India, North Korea, Pakistan, Russia, South Korea, Singapore, and Japan, likely due to socioeconomic differences [38]. Additionally, our study found that the growth trend of blindness and vision loss caused by near vision loss from 1990 to 2021 differed significantly across SDI regions. In the middle SDI region, near vision loss increased the fastest, reaching 38.7%, while it increased by 0.7% in the low SDI region and 9.1% in the high SDI region. These results indicate that the observed differences in near vision loss prevalence across SDI regions reflect population-level patterns rather than individual-level effects. Middle SDI regions showed the fastest increase in prevalence, suggesting areas where public health interventions may be particularly important. Furthermore, our study also found that, among the 204 countries globally, Côte d'Ivoire, Burkina Faso, and Benin – located in the Western sub-Saharan Africa region – had the fastest-growing ASR of DALY from 1990 to 2021. Further analysis revealed that the primary contributor to the increase in blindness and vision loss in the Western sub-Saharan Africa region was refraction disorders, which grew by 9.5%. Previous studies on vision impairment caused by onchocerciasis in Africa also showed a significant reduction in ASR from 1990 to 2021 [39]. Refraction disorders accounted for a substantial portion of the increase in ASR of DALY in West Africa during 1990-2021. These findings describe regional trends and should be interpreted as ecological observations rather than direct causal links. Research has also confirmed that refraction disorders are primarily concentrated in populations of lower socioeconomic status [40,41]. Thus, given that the specific causes of blindness and vision loss vary across regions and countries, and the corresponding epidemiological trends may differ significantly, it is necessary to develop region-specific and country-specific prevention and intervention measures for blindness and vision loss.

Our analysis also reveals a gender disparity in the burden of blindness and vision loss, with both the prevalence and DALY rates being significantly higher in females compared to males. Previous studies have also confirmed this finding [33,42]. The factors contributing to this gender difference are complex, including biological factors such as women having a longer average life expectancy than men [43,44], and being more susceptible to cataracts and age-related vision conditions [45,46]. Additionally, social factors, such as potential gender discrimination, may overestimate the burden of blindness and vision loss in women. The age subgroup results also show significant differences in the causes of blindness and vision loss across different age groups. In children and adolescents, blindness and vision loss are primarily caused by refraction disorders, while in the elderly population, near vision loss and cataracts account for the majority of cases. Therefore, for children and adolescents, recognising that myopia is mainly driven by environmental factors related to intensive learning suggests that increasing outdoor time for school-aged children is crucial. In the elderly population, strengthening community support and education, conducting regular ophthalmic check-ups for seniors, and providing cataract surgery or vision aids and rehabilitation for those in need will play an important role.

Despite its comprehensive scope, this study has several inherent limitations. First, although the GBD 2021 study employs advanced meta-regression methods, data quality and availability vary significantly across regions. In data-sparse settings, estimates rely heavily on covariate-based modelling (*e.g*. SDI), which may constrain the precision of cross-country comparisons and potentially obscure localised epidemiological patterns [47]. Second, the redistribution of unspecified causes (‘garbage codes’) into the six specific aetiologies-such as glaucoma or age-related macular degeneration-may introduce uncertainty into the proportional distribution, especially for conditions with complex diagnostic criteria. Third, regional data sparsity remains a persistent challenge; in underdeveloped areas, the burden of vision loss may be underreported due to limited diagnostic infrastructure and restricted health care access. Furthermore, studies involving children are frequently school-based, which may introduce selection bias in countries with low enrolment rates, particularly for refractive error assessments [48]. Fourth, methodological revisions across successive GBD iterations, including updates to disability weights and data mapping protocols, may influence the consistency of observed temporal trends from 1990 to 2021. Fifth, our analysis did not explicitly account for the influence of genetic factors, ethnicity, or specific lifestyle behaviours, nor did it stratify by varying severity levels of vision loss-all of which are critical for tailoring context-specific public health interventions. Regarding statistical uncertainty, while the BAPC model propagates uncertainty through its Bayesian inferential framework (INLA) to provide 95% CrIs for 2050 projections, other sequential analyses (*e.g*. Joinpoint regression and Spearman correlation) were conducted using GBD point estimates and their reported uncertainty intervals, rather than a full propagation of the 1000 posterior draws. Finally, our SDI correlation analysis is based on country-level ecological observations. This approach is limited by the non-independence of observations, as countries within the same geographic region often share similar environmental, genetic, and policy-related covariates. Consequently, the reported significance levels should be interpreted with caution, as they do not account for spatial autocorrelation or regional clustering. Future research employing mixed-effects modelling or spatial regression is warranted to more precisely quantify these complex socio-economic relationships.

## CONCLUSIONS

This study provides an updated and comprehensive assessment of the global prevalence and DALYs of blindness and vision loss through 2021, utilising the latest GBD 2021 framework. By applying Joinpoint regression and BAPC modelling, we characterised historical trend inflections and provided robust projections through 2050 across various regions, SDI levels, and demographic groups. Our findings indicate that the overall burden is driven by divergent cause-specific trends: while the burden associated with near vision loss and cataracts has shown a gradual global increase over the past 30 years, that caused by age-related macular degeneration, glaucoma, refractive errors, and other causes has declined. Notably, while near vision loss dominates prevalence numbers, its contribution to the overall DALY burden is relatively limited compared to sight-threatening conditions like cataracts. These results underscore the critical need for region-specific and cause-targeted prevention strategies. Furthermore, the projected shift in burden toward older age groups necessitates tailored health care infrastructure and intervention policies that address the specific visual needs-ranging from refractive correction to surgical treatment-predominant in different demographic and socio-economic contexts.

## Additional material


Online Supplementary Document
