# Supplementary material for: Global, regional, and national trends in blindness and vision loss, 1990–2021: a secondary ecological trend analysis based on modelled population estimates
Source: J Glob Health. 2026 May 29;16:04176. doi: 10.7189/jogh.16.04176 (PMC13220649; doi:10.7189/jogh.16.04176)
Supplement: Online Supplementary Document [file jogh-16-04176-s001.pdf]

Supplement to: Wu SN, Jiang Y, Guan W, Xu C, Zeng DX, Yan B, Lv J, Huang C, Hu J, Han Y, Liu Z. Global, regional, and national trends in blindness and vision loss, 1990–2021: a secondary ecological trend analysis based on modelled population estimates. J Glob Health. 2026;16:04176.

Table of Contents

Appendix1. Guideline 1

Supplementary table 1. Adherence to JoGH's GRABDROP guidelines items. 1

Appendix2. Supplementary results 4

Supplementary tables 4

Supplementary table 2. Trends in ASPR from 1990 to 2021 for 204 countries and regions. 4

Supplementary table 3. Trends in ASR of DALY from 1990 to 2021 for 204 countries and regions. 13

Supplementary table 4.. BAPC model predictions of blindness and vision loss and its specific causes by gender subgroups up to 2050. 22

Supplementary figures 24

Supplementary Figure 1. Global maps of AAPC results for blindness and vision loss in ASPR and ASR of DALY across 204 countries. 24

Supplementary Figure 2. Trend analysis of blindness and vision loss in ASPR and ASR of DALY by gender subgroups. 25

Supplementary Figure 3. Trend analysis of ASR of DALY for blindness and vision loss caused by different conditions from 1990 to 2021. 26

Supplementary Figure 4. Correlation analysis results between ASPR and ASR of DALY and SDI levels in 204 countries globally in 2021. 27

Supplementary Figure 5. Comparison of trends in ASPR and ASR of DALYs for blindness and vision loss and their specific causes across regions with different SDI levels globally. 28

Supplementary Figure 6. Age subgroup analysis in 2021 showing the proportion of

six different causes of blindness and vision loss in the prevalence and DALY number metrics. 29

Supplementary Figure 7. Distribution of prevalence and prevalence rates, as well as DALY counts and DALY rates, for blindness and vision loss across different age groups globally in 1990 and 2021. 30

Supplementary Figure 8. Trends in blindness and vision loss prevalence numbers and their causes globally and across 26 regions from 1990 to 2021. 31

Supplementary Figure 9. Trends in blindness and vision loss ASPR and its causes globally and across 26 regions from 1990 to 2021. 32

Supplementary Figure 10. Trends in blindness and vision loss DALY numbers and their causes globally and across 26 regions from 1990 to 2021. 33

Supplementary Figure 11. Trends in blindness and vision loss ASR of DALY and its causes globally and across 26 regions from 1990 to 2021. 34

Supplementary Figure 12. Back-testing validation of the BAPC model for global blindness and vision loss (2010–2021). 35

Supplementary Figure 13. BAPC prediction of blindness and vision loss by gender subgroups up to 2050. 36

Supplementary Figure 14. BAPC prediction of different causes of blindness and vision loss by gender subgroups up to 2050. 37

Appendix1. Guideline

Supplementary table 1. Adherence to JoGH's GRABDROP guidelines items.

**JoGH guideline items**
1. Please list all papers published by each co-author in previous 3 years that were based on secondary analysis of a big data repository
1) Wu S-N, Chen X-D, Yan D, Wang Y-Q, Wang S-P, Guan W-Y, et al. Drug-associated glaucoma: A real-world study based on the Food and Drug Administration adverse event reporting system database. Clin Exp Ophthalmol 2025;53:140–60. https://doi.org/10.1111/ceo.14454.<br>2) Wu S-N, Chen X-D, Zhang Q-H, Wang Y-Q, Yan D, Xu C-S, et al. Drug-Related Keratitis: A Real-World FDA Adverse Event Reporting System Database Study. Transl Vis Sci Technol 2024;13:17. https://doi.org/10.1167/tvst.13.9.17.<br>3) Guan W, Wu S-N, Feng K, Xu C, Liu Y, Yan B, et al. Real-world database evaluation of drug-associated vitreous opacities and machine learning for clinical interpretability. Front Cell Dev Biol 2025;13:1699669. https://doi.org/10.3389/fcell.2025.1699669.<br>4) Wu S-N, Huang C, Wang Y-Q, Chen X-D, Li X, Zhang S-Q, et al. Real-World Large Sample Assessment of Drug-related Dry Eye Risk: Based on the FDA Adverse Event Reporting System Database. Asia Pac J Ophthalmol (Phila) 2024;13:100104. https://doi.org/10.1016/j.apjo.2024.100104.<br>5) Wu S-N, Huang C, Chen X-D, Liu Q-T, Chen L, Wang S-P, et al. Retrospective cohort study of ocular adverse reactions to systemic drugs: insights from a multi-country spontaneous reporting database. Int J Surg 2026;112:2288–300. https://doi.org/10.1097/JS9.0000000000003603.<br>6) Wu S-N, Qin D-Y, Zhu L, Guo S-J, Li X, Huang C-H, et al. Uveal melanoma distant metastasis prediction system: A retrospective observational study based on machine learning. Cancer Sci 2024;115:3107–26. https://doi.org/10.1111/cas.16276.<br>7) Wu SN, Liu R, Chen L, Wang SP, Chen XD, Qin DY, Yan B, Guo SJ, Huang CH, Hu J, Liu Z. Global burden of early-onset mental disorders in adolescents and young adults: a systematic analysis based on the Global Burden of Disease study 2021. BMC Psychiatry. 2025 Nov 28;25(1):1136. https://10.1186/s12888-025-07581-x.
2. Please explain the key elements of your study design and the use of the available datasets that make your study an original scientific contribution

This study presents a comprehensive secondary analysis of the Global Burden of Disease (GBD) 2021 dataset, examining the burden of blindness and vision loss across 204 countries and territories from 1990 to 2021. Using the most up-to-date publicly available GBD 2021 data, we jointly assessed prevalence and disability-adjusted life years (DALYs), together with their age-standardized rates, across causes, sex, age, region, country, and sociodemographic index (SDI) levels. The study goes beyond a routine descriptive update by integrating several complementary analytical components, including cause-specific temporal trend analyses using Joinpoint regression, inequality assessment through SDI-stratified comparisons and Spearman correlation analyses, and future burden projections to 2050 using a Bayesian age-period-cohort model. In addition, the study provides a detailed cause-specific framework covering age-related macular degeneration, cataract, glaucoma, near vision loss, refraction disorders, and other vision loss. Therefore, the originality of this work lies in providing an updated, multidimensional, and policy-relevant global assessment of blindness and vision loss based on GBD 2021, rather than a simple replication of previous GBD-based studies.

3. Please list all publications that addressed similar research questions in the same dataset and indicate where you cited them in your paper

1) Causes of blindness and vision impairment in 2020 and trends over 30 years, and prevalence of avoidable blindness in relation to VISION 2020: the Right to Sight: an analysis for the Global Burden of Disease Study. Lancet Glob Health 2021; 9(2): e144-e160.

2) Liu L, Jiao J, Yang X, Zhang J, Yu H, Li C et al. Global, Regional, and National Burdens of Blindness and Vision Loss in Children and Adolescents from 1990 to 2019: A Trend Analysis. Ophthalmology 2023; 130(6): 575-587.

3) Xu T, Wang B, Liu H, Wang H, Yin P, Dong W et al. Prevalence and causes of vision loss in China from 1990 to 2019: findings from the Global Burden of Disease Study 2019. Lancet Public Health 2020; 5(12): e682-e691.

4) Trends in prevalence of blindness and distance and near vision impairment over 30 years: an analysis for the Global Burden of Disease Study. Lancet Glob Health 2021; 9(2): e130-e143.

5) Jin G, Zou M, Liu C, Chen A, Sun Y, Young CA et al. Burden of near vision loss in China: findings from the Global Burden of Disease Study 2019. Br J Ophthalmol 2023; 107(3): 436-441.

4. Please explain how you addressed multiple testing through an appropriately rigorous statistical threshold and indicate this in the methods section
Because this study was primarily a descriptive secondary analysis of GBD 2021 data, with prespecified outcomes and subgroup stratifications, the statistical inference was limited to trend estimation and correlation analyses rather than extensive exploratory hypothesis screening. We reported all estimates with 95% uncertainty intervals or 95% confidence intervals, and all statistical tests were two-sided with a significance threshold of $P < 0.05$. In addition, we interpreted statistical significance cautiously, with emphasis placed on the magnitude, direction, and consistency of findings across prevalence, DALYs, age-standardized rates, and subgroup analyses, rather than on isolated P values alone (see Methods, Statistical analysis section).

5. Please declare to what extent have AI chatbots been used in developing your paper and to which parts of the paper did they contribute
AI chatbots were not used in the analysis, interpretation, or writing of this manuscript. All content was developed by the authors through independent literature review, data extraction from the publicly available GBD 2021 database, statistical analysis using R and Joinpoint Regression Program, and critical interpretation of the findings.

Appendix2. Supplementary results

Supplementary tables

Supplementary table 2. Trends in ASPR from 1990 to 2021 for 204 countries and regions.

Location	1990 ASR (95%UI)	2021 ASR (95%UI)	AAPC(95%CI)	P value
Afghanistan	14563.98 (12418.28 to 17371.11)	14302.13 (12127.45 to 17043.4)	-0.02(-0.04 to -0.01)	<0.001
Albania	7804.09 (6321.31 to 9692.06)	7698.84 (6236.25 to 9509.95)	-0.06(-0.07 to -0.05)	<0.001
Algeria	13840.97 (11721.05 to 16654.9)	13348.02 (11143.08 to 16096.17)	-0.12(-0.12 to -0.12)	<0.001
American Samoa	13165.55 (10765.04 to 16190.59)	12942.51 (10561.86 to 16097.43)	-0.05(-0.06 to -0.05)	<0.001
Andorra	7466 (6019.76 to 9309.02)	7422.98 (6027.66 to 9192.25)	-0.01(-0.03 to 0)	0.029
Angola	20432.29 (16296.52 to 25920.65)	20177.13 (15994.63 to 25605.69)	-0.04(-0.05 to -0.04)	<0.001
Antigua and Barbuda	13346 (10686.07 to 16692.45)	13041.55 (10384.71 to 16402.2)	-0.08(-0.08 to -0.08)	<0.001
Argentina	9348.3 (7648.03 to 11700.04)	9189.99 (7476.28 to 11541.22)	-0.05(-0.06 to -0.04)	<0.001
Armenia	11614.93 (9703.64 to 14096.12)	11381.13 (9444.72 to 13646.85)	-0.07(-0.07 to -0.07)	<0.001
Australia	8050.68 (6388.59 to 10411.91)	8008.07 (6369.84 to 10216.91)	-0.02(-0.03 to -0.01)	0.012
Austria	7704.04 (6213.86 to 9629.25)	7521.19 (6061.18 to 9435.21)	-0.08(-0.1 to -0.07)	<0.001
Azerbaijan	11639.58 (9730.67 to 14225.48)	11410.73 (9476.96 to 13748.93)	-0.07(-0.07 to -0.06)	<0.001
Bahamas	13277.52 (10628.26 to 16584.91)	13072.26 (10394.29 to 16456.69)	-0.05(-0.06 to -0.05)	<0.001
Bahrain	13498.2 (11322.22 to 16238.48)	12884.71 (10758.7 to 15577.62)	-0.16(-0.17 to -0.15)	<0.001
Bangladesh	14770.66 (12455.28 to 18071.58)	14500.98 (11973.92 to 17846.57)	-0.05(-0.28 to 0.2)	0.62
Barbados	12278.59 (9551.88 to 15699.61)	12094.4 (9431.88 to 15551.61)	-0.04(-0.05 to -0.03)	<0.001
Belarus	10625.46 (8741.22 to 13066.98)	10489.64 (8604.53 to 13044.39)	-0.04(-0.05 to -0.04)	<0.001
Belgium	7673.98 (6174.02 to 9534.98)	7524.67 (6062.02 to 9442.38)	-0.07(-0.08 to -0.06)	<0.001
Belize	13274.97 (10742.6 to 16516.29)	13090.96 (10460.53 to 16245.72)	-0.04(-0.05 to -0.03)	<0.001
Benin	14099.49 (11129.35 to 18041.97)	14978.15 (12038.81 to 18692.48)	0.18(0.17 to 0.19)	<0.001

Bermuda	13314.13 (10624.12 to 16648.03)	13006.47 (10387.55 to 16341.78)	-0.07(-0.08 to -0.06)	<0.001
Bhutan	15369.77 (12227.72 to 19647.54)	14774.51 (11544.86 to 19031.63)	-0.15(-0.16 to -0.13)	<0.001
Bolivia (Plurinational State of)	16783.07 (13667.48 to 20892.85)	16372.16 (13249.4 to 20320.14)	-0.08(-0.09 to -0.08)	<0.001
Bosnia and Herzegovina	12832.39 (10105.06 to 16328.79)	12688.68 (10048.19 to 16048.95)	0.03(0 to 0.06)	0.068
Botswana	19942.49 (15707.39 to 25254.34)	19386.83 (15069.58 to 24726.93)	-0.09(-0.1 to -0.09)	<0.001
Brazil	17489.78 (14238.21 to 21646.26)	17223.33 (14008.89 to 21376.69)	-0.01(-0.13 to 0.12)	0.764
Brunei Darussalam	8043.1 (6381.14 to 10375.58)	7941.05 (6307.06 to 10313.14)	-0.04(-0.05 to -0.03)	<0.001
Bulgaria	7710.72 (6237.82 to 9655.19)	7641.03 (6278.82 to 9465.43)	-0.04(-0.04 to -0.03)	<0.001
Burkina Faso	8785.13 (6999.86 to 10933.08)	9875.77 (8229.24 to 11932.79)	0.51(0.42 to 0.59)	<0.001
Burundi	11678.45 (9153.73 to 14569.53)	11494.26 (9006.33 to 14472.16)	-0.06(-0.08 to -0.05)	<0.001
Cabo Verde	14565.18 (11551.38 to 18431.94)	14088.67 (11197.05 to 17802.06)	-0.11(-0.12 to -0.1)	<0.001
Cambodia	12907.11 (11298.58 to 14794.1)	11792.04 (10241.97 to 13789.51)	-0.34(-0.36 to -0.31)	<0.001
Cameroon	14412.36 (11477.32 to 18221.45)	14151.9 (11157.26 to 17890.09)	-0.08(-0.09 to -0.06)	<0.001
Canada	6696.54 (5191.71 to 8669.68)	6643.33 (5127.05 to 8677.37)	-0.03(-0.04 to -0.03)	<0.001
Central African Republic	19434.43 (15006.98 to 24944.48)	19466.98 (15080.52 to 25009.18)	0.01(-0.01 to 0.02)	0.296
Chad	17647.08 (13952.36 to 22634.77)	17801.52 (14096.3 to 22594.19)	0.49(0.27 to 0.68)	0.001
Chile	9352.86 (7688.33 to 11699.33)	9122.49 (7401.82 to 11536.98)	-0.08(-0.1 to -0.07)	<0.001
China	12068.81 (9799.7 to 14928.08)	17428.86 (13625.18 to 22177.12)	1.23(0.99 to 1.59)	<0.001
Colombia	15029.36 (13492.01 to 16938.73)	16252.55 (13189.98 to 20170.57)	0.36(0.3 to 0.42)	<0.001
Comoros	19626.33 (15714.49 to 24696.21)	19247.04 (15308.17 to 23772.65)	0.12(0.05 to 0.18)	0.003
Congo	20626.69 (16344.33 to 25937.14)	20281.52 (16074.13 to 25613.02)	0.19(0.07 to 0.3)	0.004
Cook Islands	13389.32 (11036.84 to 16514.43)	13000.72 (10618.27 to 16059.07)	-0.09(-0.09 to -0.08)	<0.001
Costa Rica	14229.99 (11678.71 to 17703.35)	13999.86 (11323.12 to 17305.77)	-0.05(-0.05 to -0.04)	<0.001
Coted'Ivoire	14974.19 (11641.44 to 19318.71)	16138.97 (12946.12 to 20235.54)	0.48(0.4 to 0.56)	<0.001
Croatia	7114.94 (5838.96 to 8663.04)	6940.38 (5674.92 to 8483.08)	0.12(0.02 to 0.22)	0.02
Cuba	13962.96 (11436.41 to 17126.67)	13747.19 (11150.75 to 17065.09)	-0.05(-0.06 to -0.05)	<0.001

Cyprus	7646.1 (6152.35 to 9591.69)	7479.94 (5994.89 to 9347.65)	-0.07(-0.08 to -0.06)	<0.001
Czechia	6920.34 (5698.15 to 8456.1)	6778.88 (5598.58 to 8313.74)	0(-0.03 to 0.04)	0.865
Democratic People's Republic of Korea	13740.97 (10743.72 to 17755.87)	13581.95 (10463.38 to 17574.5)	-0.04(-0.05 to -0.04)	<0.001
Democratic Republic of the Congo	19345.98 (15103.64 to 24950.41)	19250.22 (14992.15 to 24733.33)	-0.02(-0.03 to -0.01)	0.024
Denmark	7735.77 (6240.79 to 9685.52)	7596.53 (6128.94 to 9462.55)	-0.06(-0.06 to -0.05)	<0.001
Djibouti	12760.05 (10458.02 to 15471.78)	12389.39 (10001.69 to 15123.1)	-0.1(-0.1 to -0.09)	<0.001
Dominica	13473.98 (10832.32 to 16744.57)	12892.49 (10296.91 to 16186.69)	-0.15(-0.15 to -0.14)	<0.001
Dominican Republic	14203.21 (11644.79 to 17442.72)	13785.25 (11182.06 to 16947.09)	0.16(0.04 to 0.27)	0.015
Ecuador	15956.29 (12803 to 19905.91)	15707.18 (12577.41 to 19727.11)	0.2(0.08 to 0.31)	0.004
Egypt	13563.27 (12105.64 to 15277.75)	14810.31 (12310.28 to 18006.17)	0.34(0.29 to 0.4)	<0.001
El Salvador	15447.1 (12974.35 to 18685.05)	15046.37 (12448.03 to 18383.04)	-0.08(-0.09 to -0.08)	<0.001
Equatorial Guinea	21211.75 (16904.48 to 26576.7)	19971.11 (15761.78 to 25274.23)	-0.21(-0.23 to -0.2)	<0.001
Eritrea	13460.59 (11100.79 to 16213.24)	12985.05 (10722.15 to 15887.17)	-0.12(-0.13 to -0.12)	<0.001
Estonia	7735.47 (6295.89 to 9725.74)	7597.75 (6126.2 to 9589.99)	0.16(0.05 to 0.26)	0.009
Eswatini	19734.03 (15439.85 to 25175.78)	19228.6 (14872.62 to 24472.64)	0.35(0.15 to 0.52)	0.004
Ethiopia	13543.64 (11110.54 to 16593.18)	13611.63 (11122.69 to 16608.74)	0.2(0.08 to 0.32)	0.004
Fiji	13603.54 (11256 to 16711.55)	13301.98 (10962.35 to 16433.5)	-0.08(-0.09 to -0.06)	<0.001
Finland	7703.41 (6206.54 to 9635.56)	7499.24 (6046.22 to 9312.11)	-0.09(-0.1 to -0.08)	<0.001
France	7295.35 (5783.93 to 9177.75)	7179.59 (5679.63 to 9073.33)	-0.06(-0.06 to -0.05)	<0.001
Gabon	20273.16 (16076.85 to 25782.48)	20017.17 (15701.41 to 25429.54)	-0.04(-0.05 to -0.02)	<0.001
Gambia	15014.71 (12097.68 to 18729.5)	14946.09 (12092.64 to 18629.76)	0(-0.03 to 0.03)	0.992
Georgia	12775.55 (11403.14 to 14211.61)	11854.77 (9782.02 to 14348.82)	-0.26(-0.35 to -0.17)	<0.001
Germany	7650.97 (6112.25 to 9604.58)	7441.07 (5961.62 to 9316.03)	-0.09(-0.1 to -0.08)	<0.001
Ghana	12585.97 (10197.25 to 15702.46)	13210.39 (10856.85 to 16193.4)	0.32(0.03 to 0.81)	0.025
Greece	7686.64 (6206.65 to 9549.67)	7571.8 (6080.9 to 9449.15)	-0.05(-0.06 to -0.04)	<0.001
Greenland	6731 (5235.15 to 8674.36)	6688.95 (5173.19 to 8651.07)	-0.03(-0.03 to -0.02)	<0.001

Grenada	13510.3 (10911.58 to 16821.61)	12974.9 (10359.37 to 16295.64)	-0.13(-0.13 to -0.13)	<0.001
Guam	13074.04 (10711.09 to 16170.53)	12964.58 (10501.36 to 16065.36)	-0.03(-0.05 to -0.02)	<0.001
Guatemala	15344.97 (12836.31 to 18523.42)	14948.58 (12403.13 to 18370.55)	0.21(0.08 to 0.32)	0.005
Guinea	14672.03 (11706.19 to 18569.45)	14973.9 (12055.9 to 18642.16)	0.06(0.05 to 0.07)	<0.001
Guinea-Bissau	14803.56 (11912.65 to 18485.94)	15020.63 (12116.76 to 18669.95)	0.05(0.04 to 0.06)	<0.001
Guyana	13418.02 (10850.85 to 16590.45)	13158.34 (10561.45 to 16503.34)	-0.06(-0.07 to -0.06)	<0.001
Haiti	13741.64 (11138.37 to 17044.33)	13448.88 (10780.1 to 16823.07)	-0.06(-0.07 to -0.05)	<0.001
Honduras	14665.63 (12076.02 to 17866.96)	14391.66 (11693.89 to 17734.63)	-0.06(-0.06 to -0.05)	<0.001
Hungary	6667.26 (5518.53 to 8163.01)	6522.92 (5396.68 to 7915.71)	0.14(0.05 to 0.22)	0.005
Iceland	7499.29 (6027.37 to 9371.43)	7379.04 (5971.26 to 9237.03)	-0.06(-0.07 to -0.06)	<0.001
India	19125.79 (16070.44 to 22994.75)	26333.59 (21116.25 to 32187.03)	1.35(1.01 to 1.9)	<0.001
Indonesia	14787.92 (12181.28 to 17838.27)	15664.32 (12673.69 to 19308.25)	0.25(0.19 to 0.31)	<0.001
Iran (Islamic Republic of)	12544.89 (10813.33 to 14845.14)	12691.46 (10732.96 to 15108.03)	0.05(0.01 to 0.09)	0.018
Iraq	13838.54 (11613.92 to 16653.31)	13304.31 (11145.17 to 16008.3)	-0.14(-0.15 to -0.13)	<0.001
Ireland	7633.48 (6144.08 to 9506.15)	7483.6 (6058 to 9342.2)	-0.07(-0.09 to -0.06)	<0.001
Israel	7629.56 (6108.48 to 9512.35)	7521.51 (6049.54 to 9379.5)	-0.05(-0.05 to -0.04)	<0.001
Italy	9269.47 (7686.75 to 11397.79)	9030.68 (7416.31 to 11117.13)	-0.09(-0.1 to -0.09)	<0.001
Jamaica	13283 (10726.4 to 16634.78)	13039.69 (10402.6 to 16493.16)	-0.07(-0.07 to -0.06)	<0.001
Japan	8463.37 (6585.21 to 10924.61)	8394.55 (6540.05 to 10943.11)	-0.04(-0.04 to -0.03)	<0.001
Jordan	13052.16 (10811.88 to 15897.81)	12627.53 (10392.92 to 15560.92)	-0.11(-0.13 to -0.1)	<0.001
Kazakhstan	11179.08 (9399.98 to 13552.04)	10963.68 (9073.38 to 13298.22)	0.13(0.05 to 0.21)	0.004
Kenya	15481.17 (12658.07 to 19036.5)	15105.43 (12227.26 to 18657.75)	-0.03(-0.07 to 0.02)	0.194
Kiribati	13507.75 (11118.73 to 16557.43)	13279.26 (10874.38 to 16434.48)	-0.06(-0.06 to -0.05)	<0.001
Kuwait	13091.7 (10977.18 to 15760.08)	12881.36 (10652.53 to 15585.29)	-0.05(-0.06 to -0.05)	<0.001
Kyrgyzstan	11154.31 (9168.17 to 13624.89)	10962.62 (8943.87 to 13456.07)	-0.06(-0.06 to -0.05)	<0.001
Lao People's Democratic Republic	8835.23 (7433.95 to 10611.78)	8548.16 (7133.28 to 10256.33)	0.01(-0.05 to 0.05)	0.845

Latvia	11432.72 (9374.43 to 14045.03)	11326.38 (9245.56 to 14075.93)	0.13(0.06 to 0.19)	0.002
Lebanon	12346.32 (11158.15 to 13688.79)	12616.1 (10623.02 to 15143.83)	0.14(0.1 to 0.18)	<0.001
Lesotho	20100.6 (15644.33 to 25277.37)	19682.72 (15483.45 to 24921.98)	-0.07(-0.07 to -0.07)	<0.001
Liberia	14927.28 (12051.01 to 18675.44)	14543.98 (11643.99 to 18218.45)	-0.09(-0.1 to -0.08)	<0.001
Libya	13769.12 (11603.97 to 16472.89)	13353.17 (11131.13 to 16275)	-0.09(-0.1 to -0.09)	<0.001
Lithuania	10596.13 (8699.33 to 12951.7)	10420.75 (8506.55 to 12970.14)	-0.05(-0.06 to -0.05)	<0.001
Luxembourg	7658.49 (6132.69 to 9622.52)	7467.49 (5994.4 to 9366.08)	-0.09(-0.1 to -0.08)	<0.001
Madagascar	12547.22 (10183.87 to 15419.48)	12359.82 (9913.56 to 15318.43)	-0.05(-0.06 to -0.05)	<0.001
Malawi	9230.12 (7895.88 to 10886.64)	8754.43 (7421.2 to 10383.64)	-0.17(-0.25 to -0.09)	<0.001
Malaysia	8561.79 (7595.43 to 9616.84)	7705.07 (6754.8 to 8827.8)	-0.22(-0.27 to -0.17)	<0.001
Maldives	10563.92 (8938.79 to 12643.65)	10008.71 (8378.09 to 12057.99)	-0.17(-0.18 to -0.16)	<0.001
Mali	11474.34 (9437.03 to 14220.47)	11808.99 (9707.19 to 14484.68)	0.28(0.21 to 0.35)	<0.001
Malta	7656.96 (6143.61 to 9628.59)	7516.96 (6040.07 to 9418.04)	-0.07(-0.08 to -0.06)	<0.001
Marshall Islands	13423.37 (11061.22 to 16517.52)	13089.07 (10660.58 to 16160.62)	-0.08(-0.09 to -0.07)	<0.001
Mauritania	21168.84 (16899.03 to 26665.25)	20821.64 (16517.94 to 26162.07)	0.26(0.12 to 0.38)	0.002
Mauritius	10422.22 (8959.13 to 12346.68)	9964.8 (8438.8 to 11889.18)	0.04(-0.04 to 0.12)	0.282
Mexico	12060.48 (10053.72 to 14496.12)	13738.62 (11114.92 to 16943.77)	0.85(0.66 to 1.03)	<0.001
Micronesia (Federated States of)	13404.88 (11077.27 to 16554.72)	13124.39 (10668.32 to 16201.51)	-0.06(-0.07 to -0.06)	<0.001
Monaco	7579.09 (6091.96 to 9415.37)	7467.19 (6026.17 to 9245)	-0.05(-0.06 to -0.04)	<0.001
Mongolia	11847.39 (9907.98 to 14315.62)	11594.5 (9646.34 to 14002.93)	-0.08(-0.08 to -0.07)	<0.001
Montenegro	7707.4 (6303.02 to 9582.87)	7618.94 (6141.89 to 9511.11)	-0.04(-0.05 to -0.04)	<0.001
Morocco	16420.48 (13341.05 to 20439.44)	16204.56 (13229.28 to 20167.06)	0.04(-0.01 to 0.08)	0.073
Mozambique	12619.38 (10214.91 to 15418.67)	12770.52 (10390.32 to 15732.88)	0.04(0.03 to 0.04)	<0.001
Myanmar	10148.92 (9054.85 to 11353.86)	9455.36 (8331.13 to 10852.14)	-0.17(-0.2 to -0.15)	<0.001
Namibia	17938.43 (14212.56 to 22910.07)	17709.64 (13837.63 to 22859.32)	0.45(0.23 to 0.65)	0.002
Nauru	13200.59 (10857.44 to 16272.03)	13138.19 (10682.26 to 16337.47)	-0.02(-0.02 to -0.01)	<0.001

Nepal	20515.7 (16427.15 to 25498.41)	25663.58 (21476.47 to 29336.61)	0.91(0.51 to 1.64)	<0.001
Netherlands	7434 (5877.42 to 9371.55)	7306.12 (5846.82 to 9195.26)	-0.06(-0.07 to -0.06)	<0.001
New Zealand	8894.71 (7037.53 to 11295.81)	8779.21 (6956.88 to 11199.76)	-0.04(-0.04 to -0.03)	<0.001
Nicaragua	14894.89 (12377.94 to 18096.43)	14487.23 (11838.42 to 17880.84)	-0.08(-0.09 to -0.08)	<0.001
Niger	19999.91 (15790.54 to 25489.98)	23557.38 (19626.43 to 27185.74)	0.74(0.48 to 0.88)	<0.001
Nigeria	16960.68 (13833.81 to 21434.78)	16928.33 (13815.68 to 21337.01)	-0.02(-0.03 to -0.01)	<0.001
Niue	13309.38 (10958.84 to 16367.67)	12987.95 (10611.5 to 16174.74)	-0.08(-0.09 to -0.08)	<0.001
North Macedonia	7758.13 (6255.02 to 9651.87)	7632.69 (6195.4 to 9480.13)	-0.05(-0.06 to -0.05)	<0.001
Northern Mariana Islands	12962.63 (10527.76 to 15970.83)	12889.85 (10543.74 to 15938.94)	-0.02(-0.03 to -0.02)	<0.001
Norway	8231.43 (6586.67 to 10323.5)	8129.99 (6522.14 to 10232.39)	-0.05(-0.05 to -0.04)	<0.001
Oman	14743.04 (12623.78 to 17590)	15143.19 (13145.03 to 17709.92)	0.07(0.05 to 0.1)	<0.001
Pakistan	14064.09 (12007.88 to 16826.14)	13864.54 (11823.24 to 16696.95)	0.09(0.03 to 0.16)	0.007
Palau	13141.67 (10723.95 to 16125.69)	12894.47 (10547.62 to 15991.87)	-0.06(-0.07 to -0.06)	<0.001
Palestine	13956.48 (11739.5 to 16763.35)	13263.97 (11033.35 to 16176.65)	-0.17(-0.18 to -0.17)	<0.001
Panama	14789.27 (12255.35 to 17920.56)	14450.57 (11863.86 to 17731.4)	-0.07(-0.08 to -0.07)	<0.001
Papua New Guinea	14442.83 (12154.03 to 17391.93)	14201.18 (11857.49 to 17285.04)	-0.11(-0.14 to -0.09)	<0.001
Paraguay	17148.55 (14076.63 to 20906.13)	16764.7 (13645.48 to 20556.05)	0.23(0.09 to 0.35)	0.005
Peru	17365.2 (14343.57 to 21352.53)	16888.54 (13796.73 to 20751.88)	-0.11(-0.13 to -0.09)	<0.001
Philippines	21129.86 (16982.02 to 26328.67)	20961.03 (16773.89 to 26115.14)	-0.01(-0.02 to 0)	0.178
Poland	8567 (6898.68 to 10599.79)	8449.14 (6757.41 to 10597.09)	-0.06(-0.06 to -0.05)	<0.001
Portugal	7739.45 (6244.41 to 9622.82)	7593.76 (6102.83 to 9433.2)	-0.06(-0.06 to -0.06)	<0.001
Puerto Rico	13361.09 (10706.3 to 16612.29)	13093.65 (10408.28 to 16501.32)	-0.06(-0.06 to -0.05)	<0.001
Qatar	13194.12 (11214.81 to 15697.71)	12652.87 (10522.63 to 15191.85)	-0.14(-0.15 to -0.13)	<0.001
Republic of Korea	8221.99 (6560.88 to 10521.5)	7971.28 (6361.98 to 10250.63)	-0.11(-0.12 to -0.1)	<0.001
Republic of Moldova	11314.08 (9412.35 to 13626.74)	11360.68 (9436.54 to 13800.15)	0.03(0.02 to 0.05)	0.001
Romania	7779.6 (6341.91 to 9725.73)	7671.61 (6294.9 to 9490.74)	-0.05(-0.06 to -0.04)	<0.001

Russian Federation	15480.32 (12550.03 to 19062.32)	18546.03 (14692.66 to 23360.4)	0.86(0.64 to 1.11)	<0.001
Rwanda	12290.82 (9850.75 to 15325.98)	12015.41 (9548.24 to 14985.85)	-0.08(-0.08 to -0.07)	<0.001
Saint Kitts and Nevis	13372.59 (10745.84 to 16629.8)	12941.2 (10358.86 to 16317.42)	-0.1(-0.1 to -0.1)	<0.001
Saint Lucia	13399.47 (10882.18 to 16579.41)	12997.56 (10310.19 to 16375.01)	-0.1(-0.11 to -0.1)	<0.001
Saint Vincent and the Grenadines	13429.88 (10764.67 to 16632.06)	12947.06 (10341.17 to 16180.78)	-0.11(-0.11 to -0.11)	<0.001
Samoa	13320.67 (10885.78 to 16475.38)	13060.82 (10602.47 to 16116.03)	-0.06(-0.07 to -0.06)	<0.001
San Marino	7558.49 (6066.67 to 9455.4)	7470.55 (6025.56 to 9356.19)	-0.04(-0.05 to -0.04)	<0.001
Sao Tome and Principe	15186.28 (12305.43 to 19026.4)	14727.16 (11793.03 to 18447.68)	-0.11(-0.11 to -0.1)	<0.001
Saudi Arabia	15610.74 (13564.3 to 18328.23)	14166.79 (12081.29 to 16874.45)	-0.32(-0.34 to -0.3)	<0.001
Senegal	13895.19 (11253.82 to 17050.46)	13487.19 (10893.26 to 16760.49)	0.2(0.07 to 0.31)	0.005
Serbia	7753.99 (6272.79 to 9707.12)	7640.02 (6163.97 to 9447.96)	-0.06(-0.09 to -0.04)	<0.001
Seychelles	10920.97 (9287.16 to 12912.6)	10508.68 (8869.79 to 12628.97)	-0.13(-0.13 to -0.13)	<0.001
Sierra Leone	14566.01 (11622.88 to 18321.94)	14315.42 (11463.39 to 18041.02)	-0.06(-0.07 to -0.06)	<0.001
Singapore	8615.25 (6942.69 to 10876.7)	8449.31 (6834.84 to 10735.62)	-0.06(-0.07 to -0.05)	<0.001
Slovakia	6605.31 (5596.23 to 7650.42)	6231.34 (5124.35 to 7594.61)	-0.2(-0.26 to -0.13)	<0.001
Slovenia	8792.02 (6983.74 to 10988.57)	8777.38 (6955.82 to 10998.87)	0.12(0.07 to 0.18)	<0.001
Solomon Islands	13433.33 (11069.67 to 16529.45)	13268.08 (10911.89 to 16310.95)	-0.04(-0.04 to -0.03)	<0.001
Somalia	12075.94 (9702.26 to 15031.7)	12071.53 (9677.74 to 15051.03)	0(-0.01 to 0.01)	0.934
South Africa	28907.89 (22879.94 to 35976.15)	29635.03 (23783.29 to 36779.82)	0.37(0.12 to 0.85)	0.003
South Sudan	13143.7 (10876.41 to 15842.96)	12836.55 (10552.1 to 15630.62)	-0.07(-0.09 to -0.06)	<0.001
Spain	9188.97 (7704.7 to 11136.78)	9064.96 (7643.96 to 10859.49)	0.06(-0.07 to 0.18)	0.358
Sri Lanka	12093.42 (10360.81 to 14336.48)	11800.86 (10046.82 to 14011.81)	-0.06(-0.11 to 0.02)	0.101
Sudan	13911.14 (11745.99 to 16724.16)	13163.18 (10952.26 to 16077.56)	-0.18(-0.19 to -0.17)	<0.001
Suriname	13666.54 (11172.68 to 16805.34)	13446.1 (10820.27 to 16570.27)	-0.06(-0.07 to -0.05)	<0.001
Sweden	7301.31 (5619.73 to 9553.27)	7245.61 (5602.55 to 9398.8)	-0.03(-0.04 to -0.02)	<0.001
Switzerland	7570.5 (6097.56 to 9369.75)	7461.5 (6032.18 to 9374.18)	-0.05(-0.06 to -0.05)	<0.001

Syrian Arab Republic	11476.47 (10444.11 to 12806.84)	11705.18 (9924.98 to 13985.35)	0.14(0.1 to 0.18)	<0.001
Taiwan (Province of China)	13238.3 (10229.82 to 17247.16)	13200.54 (10173.35 to 17089.73)	-0.01(-0.02 to 0)	0.005
Tajikistan	11638.92 (9680.63 to 14170.47)	11440.74 (9541.1 to 13929.03)	-0.06(-0.07 to -0.05)	<0.001
Thailand	11603.06 (9996.69 to 13634.5)	11004.01 (9359.25 to 13032.14)	-0.18(-0.21 to -0.16)	<0.001
Timor-Leste	12219.31 (10666.86 to 14170.31)	11526.11 (10016.55 to 13413.86)	-0.15(-0.17 to -0.13)	<0.001
Togo	14649.87 (11646.32 to 18492.66)	14914.16 (12026.29 to 18616.3)	0.05(0.04 to 0.07)	<0.001
Tokelau	13408.92 (11016.75 to 16535.55)	13063.29 (10660.57 to 16137.66)	-0.09(-0.1 to -0.08)	<0.001
Tonga	12646.62 (10147.11 to 15970.82)	12445.55 (9982.46 to 15634.38)	-0.05(-0.06 to -0.04)	<0.001
Trinidad and Tobago	13250.55 (10653.15 to 16473.02)	12968.98 (10339.86 to 16359.86)	-0.07(-0.07 to -0.06)	<0.001
Tunisia	12910.2 (10758.22 to 15714.65)	12379.55 (10136.63 to 15132.78)	0.22(0.06 to 0.37)	0.016
Turkey	12509.09 (10562.13 to 15025.9)	14326.6 (11608.75 to 17834.17)	0.39(0.25 to 0.5)	<0.001
Turkmenistan	12506.66 (10611.55 to 14873.56)	11965.61 (10013.84 to 14224.63)	-0.17(-0.19 to -0.16)	<0.001
Tuvalu	13613.09 (11238.07 to 16777.33)	13155.01 (10773.46 to 16213.79)	-0.11(-0.11 to -0.1)	<0.001
Uganda	11798.25 (9286.73 to 14694.31)	12060.97 (9594.51 to 15010.71)	0.09(0.08 to 0.09)	<0.001
Ukraine	10537.05 (8628.02 to 12854.26)	10407.83 (8522.47 to 12729.21)	0.07(0.01 to 0.13)	0.02
United Arab Emirates	12784.19 (10823.23 to 15195.21)	11869.64 (9965.08 to 14327.75)	-0.23(-0.31 to -0.14)	<0.001
United Kingdom	8522.93 (6901.61 to 10653.02)	8363.51 (6771.9 to 10494.92)	-0.06(-0.08 to -0.06)	<0.001
United Republic of Tanzania	13278.17 (10928.75 to 16135.05)	12895.48 (10561.55 to 15586.61)	-0.1(-0.12 to -0.09)	<0.001
United States of America	6757.82 (5326.04 to 8685.9)	7327.32 (5618.19 to 9675.91)	0.87(0.6 to 1.13)	<0.001
United States Virgin Islands	13385.71 (10711.81 to 16629.83)	13045.8 (10405.1 to 16388.63)	-0.07(-0.08 to -0.06)	<0.001
Uruguay	9041.33 (7361.31 to 11376.16)	8919.14 (7164.19 to 11268.92)	0.17(0.06 to 0.27)	0.006
Uzbekistan	11644.44 (9694.12 to 14091.88)	11390.95 (9428.51 to 13851.04)	-0.08(-0.08 to -0.07)	<0.001
Vanuatu	12938.59 (10482.74 to 15982.56)	12869.17 (10379.93 to 16089.76)	-0.02(-0.02 to -0.01)	<0.001
Venezuela (Bolivarian Republic of)	14317.38 (11753.03 to 17674.61)	14143.57 (11520.29 to 17540.55)	-0.05(-0.05 to -0.04)	<0.001
Viet Nam	9012.84 (7877.42 to 10377.93)	8424.76 (7244.68 to 9947.63)	-0.15(-0.19 to -0.11)	<0.001
Yemen	12878.8 (10630.24 to 15788.78)	13207.46 (10846.11 to 16076.23)	0.08(0.07 to 0.08)	<0.001

Zambia	11929.97 (9725.34 to 14539.54)	11627.21 (9395.87 to 14270.46)	0.21(0.06 to 0.34)	0.012
Zimbabwe	15669.78 (12386.62 to 20065.73)	15456.42 (12197.72 to 19839)	0.39(0.16 to 0.6)	0.004

Abbreviations: ASR, age-standardized rate; AAPC, average annual percent change; UI, uncertainty interval; ASPR, age-standardized prevalence rate.

Supplementary table 3. Trends in ASR of DALY from 1990 to 2021 for 204 countries and regions.

Location	1990 ASR (95%UI)	2021 ASR (95%UI)	AAPC(95%CI)	P value
Afghanistan	605.15 (434.37 to 831.42)	522.64 (379.08 to 721.99)	-0.29(-0.36 to -0.23)	<0.001
Albania	180 (120.56 to 260.79)	164.22 (106.31 to 242.45)	-0.34(-0.36 to -0.31)	<0.001
Algeria	486.06 (345.42 to 675.22)	385.7 (271.26 to 540.36)	-0.76(-0.77 to -0.75)	<0.001
American Samoa	319.29 (217.09 to 463.75)	277.78 (179.49 to 409.33)	-0.45(-0.46 to -0.43)	<0.001
Andorra	159.84 (104.49 to 234.99)	151.43 (97 to 224.95)	-0.16(-0.17 to -0.16)	<0.001
Angola	472.18 (316.88 to 686.31)	399.89 (252.49 to 613.42)	-0.55(-0.56 to -0.54)	<0.001
Antigua and Barbuda	291.98 (193.6 to 429.32)	253.4 (160.77 to 381.59)	-0.46(-0.47 to -0.46)	<0.001
Argentina	222.68 (147.55 to 323.82)	201.71 (129.59 to 299.11)	-0.3(-0.33 to -0.27)	<0.001
Armenia	324.19 (224.01 to 467.55)	285.15 (193.43 to 419.16)	-0.44(-0.46 to -0.42)	<0.001
Australia	159.66 (100.95 to 239.55)	153.79 (95.78 to 234.64)	-0.09(-0.12 to -0.06)	<0.001
Austria	167.08 (111.27 to 246.26)	153.67 (99.14 to 229.65)	-0.27(-0.28 to -0.26)	<0.001
Azerbaijan	331.36 (228.75 to 480.61)	294.65 (201.97 to 428.68)	-0.4(-0.43 to -0.37)	<0.001
Bahamas	285.68 (187.26 to 419.92)	255.19 (161.34 to 386.74)	-0.4(-0.41 to -0.38)	<0.001
Bahrain	454.16 (322.52 to 626.61)	349.74 (243.37 to 495.33)	-0.85(-0.87 to -0.84)	<0.001
Bangladesh	542.56 (391.75 to 746.03)	426.41 (296.39 to 602.94)	-0.71(-0.8 to -0.61)	<0.001
Barbados	192.94 (114.17 to 312.28)	180.66 (103.65 to 298.87)	-0.2(-0.21 to -0.19)	<0.001
Belarus	254.71 (172.31 to 368.82)	231.72 (151.4 to 341.78)	-0.32(-0.33 to -0.31)	<0.001
Belgium	168.86 (112.12 to 249.57)	154.69 (101.14 to 231.26)	-0.29(-0.29 to -0.28)	<0.001
Belize	319.81 (218.49 to 459.76)	273.28 (178.36 to 403.47)	-0.49(-0.5 to -0.48)	<0.001
Benin	358.08 (245.95 to 508.74)	433.73 (305.82 to 612.13)	0.59(0.55 to 0.65)	<0.001
Bermuda	281.57 (182.65 to 414.53)	247.74 (154.57 to 373.72)	-0.41(-0.42 to -0.41)	<0.001
Bhutan	399.63 (274.13 to 574.99)	311.02 (201.97 to 467.11)	-0.86(-0.9 to -0.83)	<0.001
Bolivia (Plurinational State of)	493.88 (341.67 to 701.32)	405.15 (273.83 to 588.85)	-0.64(-0.65 to -0.64)	<0.001

Bosnia and Herzegovina	230.54 (143.02 to 358.42)	210.72 (125.93 to 339.21)	-0.31(-0.33 to -0.29)	<0.001
Botswana	529.38 (364.44 to 753.15)	416.59 (277.54 to 612.64)	-0.8(-0.81 to -0.78)	<0.001
Brazil	474.4 (328.15 to 674)	405.83 (271.87 to 592.83)	-0.42(-0.52 to -0.32)	<0.001
Brunei Darussalam	174.12 (114.25 to 257.73)	159 (101.38 to 240.1)	-0.26(-0.28 to -0.25)	<0.001
Bulgaria	161.9 (105.52 to 243.1)	152.37 (96.83 to 232.09)	-0.19(-0.2 to -0.18)	<0.001
Burkina Faso	259.89 (183.4 to 361.83)	341.71 (245.32 to 472.56)	0.82(0.73 to 0.93)	<0.001
Burundi	239.6 (158.16 to 355.92)	226.41 (148.62 to 338.19)	-0.26(-0.3 to -0.23)	<0.001
Cabo Verde	381.17 (263.9 to 539.84)	317.01 (212.29 to 469.04)	-0.62(-0.65 to -0.6)	<0.001
Cambodia	633.7 (464.47 to 855.73)	431.74 (311.64 to 605.47)	-1.34(-1.42 to -1.26)	<0.001
Cameroon	363.66 (252.07 to 526.03)	325.28 (220.12 to 475.78)	-0.41(-0.45 to -0.36)	<0.001
Canada	130.68 (81.65 to 198.65)	126.3 (78.54 to 194.39)	-0.11(-0.11 to -0.1)	<0.001
Central African Republic	313.98 (192.4 to 508.36)	326.65 (204.2 to 519.22)	0.15(0.13 to 0.18)	<0.001
Chad	441.19 (302.39 to 624.76)	442.73 (301.62 to 630.24)	0.11(0.05 to 0.18)	0.002
Chile	224.11 (149.33 to 324.57)	194.74 (124.44 to 285.79)	-0.45(-0.46 to -0.44)	<0.001
China	297.23 (204.65 to 431.01)	323.86 (203.78 to 504.04)	0.49(0.28 to 0.68)	0.003
Colombia	418.52 (294.83 to 584.68)	363.49 (236.85 to 541.57)	-0.41(-0.44 to -0.37)	<0.001
Comoros	482.42 (330.78 to 687.8)	405.14 (267.99 to 601.24)	-0.49(-0.53 to -0.45)	<0.001
Congo	431.29 (275.92 to 646.03)	382.09 (233.82 to 592)	-0.32(-0.36 to -0.27)	<0.001
Cook Islands	383.9 (268.95 to 548.93)	302.52 (201.74 to 440.92)	-0.76(-0.78 to -0.75)	<0.001
Costa Rica	363.18 (248.18 to 522.68)	310.48 (203.25 to 453.33)	-0.51(-0.51 to -0.5)	<0.001
Coted'Ivoire	283.46 (184.1 to 433.74)	408.76 (282.28 to 584.18)	1.19(1.08 to 1.3)	<0.001
Croatia	157.06 (102.74 to 230.8)	146.29 (95.21 to 217.67)	-0.16(-0.19 to -0.13)	<0.001
Cuba	369.93 (254.94 to 531.02)	321.32 (215.48 to 471.92)	-0.45(-0.46 to -0.44)	<0.001
Cyprus	170.42 (112.56 to 249.32)	153.26 (98.62 to 230.63)	-0.33(-0.34 to -0.31)	<0.001
Czechia	154.14 (101.98 to 225.82)	141.39 (91.34 to 210.83)	-0.24(-0.26 to -0.22)	<0.001
Democratic People's Republic of Korea	220.65 (128.06 to 362.64)	198.44 (109.8 to 339.96)	-0.35(-0.36 to -0.34)	<0.001

Democratic Republic of the Congo	286.93 (166.27 to 472.5)	292.65 (170.34 to 480.72)	0.05(0.04 to 0.06)	<0.001
Denmark	168.33 (109.94 to 247.89)	158 (101.52 to 237.3)	-0.2(-0.21 to -0.19)	<0.001
Djibouti	408.73 (290.81 to 572.25)	333.53 (233.13 to 476.03)	-0.64(-0.65 to -0.63)	<0.001
Dominica	303.2 (202.07 to 443.25)	259.19 (166.26 to 389.11)	-0.51(-0.52 to -0.51)	<0.001
Dominican Republic	383.69 (264.08 to 552.59)	321.45 (214.39 to 467.73)	-0.48(-0.52 to -0.43)	<0.001
Ecuador	376.27 (251.76 to 550.19)	328.93 (213.13 to 492.37)	-0.39(-0.43 to -0.35)	<0.001
Egypt	440.89 (310.19 to 615.14)	418.25 (290.46 to 593.95)	-0.16(-0.27 to -0.06)	0.001
El Salvador	498.22 (346.64 to 694.55)	389.13 (266 to 565.21)	-0.82(-0.83 to -0.8)	<0.001
Equatorial Guinea	705.01 (496.43 to 973.73)	438.9 (292.51 to 642.12)	-1.67(-1.77 to -1.59)	<0.001
Eritrea	523.4 (372.03 to 718.94)	416.84 (296.64 to 576.41)	-0.74(-0.75 to -0.72)	<0.001
Estonia	174.94 (117.69 to 255.82)	158.91 (104.23 to 236.38)	-0.24(-0.27 to -0.21)	<0.001
Eswatini	498.78 (345.54 to 711.16)	414.1 (275.81 to 610.6)	-0.57(-0.63 to -0.53)	<0.001
Ethiopia	470.37 (336.77 to 637.18)	449.95 (320.99 to 617.8)	-0.09(-0.13 to -0.05)	<0.001
Fiji	394.26 (276.26 to 558.4)	333.31 (228.73 to 484.02)	-0.55(-0.64 to -0.5)	<0.001
Finland	167.58 (110.28 to 244.69)	150.65 (95.46 to 223.99)	-0.35(-0.35 to -0.34)	<0.001
France	156.52 (104.03 to 230.38)	144.66 (93.27 to 218.01)	-0.26(-0.28 to -0.24)	<0.001
Gabon	429.33 (277.71 to 633.89)	370.67 (227.85 to 579.39)	-0.48(-0.5 to -0.46)	<0.001
Gambia	441.01 (311.33 to 612.37)	397.59 (274.01 to 563.98)	-0.29(-0.4 to -0.17)	<0.001
Georgia	324.24 (224.94 to 470.23)	294.29 (200.13 to 429.63)	-0.29(-0.32 to -0.26)	<0.001
Germany	162.16 (106.46 to 239.24)	149.27 (94.84 to 224.8)	-0.27(-0.28 to -0.27)	<0.001
Ghana	317.4 (215.3 to 453.45)	342.18 (233.12 to 488.63)	0.32(0.26 to 0.37)	<0.001
Greece	173.78 (114.77 to 253.72)	157.86 (101.21 to 232.78)	-0.3(-0.31 to -0.29)	<0.001
Greenland	139.78 (89.8 to 207.33)	133.22 (84.71 to 201.28)	-0.16(-0.17 to -0.15)	<0.001
Grenada	317.63 (215.37 to 458.71)	262.08 (169.55 to 391.87)	-0.61(-0.62 to -0.6)	<0.001
Guam	312.52 (208.17 to 458.19)	284 (183.17 to 420.06)	-0.34(-0.36 to -0.31)	<0.001
Guatemala	508.61 (357.26 to 711.07)	411.25 (285.71 to 586.03)	-0.63(-0.67 to -0.58)	<0.001

Guinea	421.89 (297.2 to 590.61)	423.91 (297.88 to 591.91)	-0.01(-0.04 to 0.03)	0.603
Guinea-Bissau	425.62 (294.49 to 598.74)	417.5 (290.31 to 590.69)	-0.04(-0.09 to 0.01)	0.121
Guyana	325.93 (222.05 to 469.07)	271.19 (177.58 to 402.02)	-0.57(-0.58 to -0.55)	<0.001
Haiti	377.27 (262.74 to 537.05)	315.76 (214.64 to 454.61)	-0.58(-0.59 to -0.56)	<0.001
Honduras	406.31 (281.51 to 580.94)	336.82 (224.47 to 492.07)	-0.61(-0.62 to -0.59)	<0.001
Hungary	173.61 (121.65 to 248.19)	151.81 (102.13 to 222.14)	-0.34(-0.38 to -0.31)	<0.001
Iceland	169.35 (112.97 to 248.05)	155.29 (101.83 to 231.2)	-0.29(-0.3 to -0.29)	<0.001
India	734.69 (527.49 to 1004.85)	632.16 (421.05 to 912.77)	-0.44(-0.56 to -0.25)	<0.001
Indonesia	477.01 (335.76 to 663.32)	399.91 (277.83 to 573.7)	-0.59(-0.64 to -0.54)	<0.001
Iran (Islamic Republic of)	528.86 (381.78 to 728.71)	448.43 (321.14 to 618.32)	-0.65(-0.72 to -0.57)	<0.001
Iraq	495.87 (353.18 to 682.31)	378.81 (265.19 to 534)	-0.9(-0.92 to -0.88)	<0.001
Ireland	168.65 (111.08 to 247.24)	153.26 (98.52 to 227.88)	-0.32(-0.33 to -0.31)	<0.001
Israel	169.61 (112.67 to 248.94)	157.26 (101.3 to 235.35)	-0.24(-0.24 to -0.23)	<0.001
Italy	262.56 (182.12 to 372.83)	224.76 (152.92 to 323.56)	-0.52(-0.54 to -0.5)	<0.001
Jamaica	297.22 (195.9 to 436.6)	260.27 (165.63 to 391.1)	-0.43(-0.44 to -0.42)	<0.001
Japan	161.67 (101.7 to 245.21)	154.13 (95.52 to 237.95)	-0.17(-0.18 to -0.15)	<0.001
Jordan	390.63 (275.13 to 546.13)	313.19 (212.65 to 453.22)	-0.73(-0.76 to -0.69)	<0.001
Kazakhstan	314.73 (220.29 to 451.9)	280.69 (192.74 to 406.92)	-0.36(-0.39 to -0.32)	<0.001
Kenya	507.63 (362.31 to 701.01)	404.1 (283.72 to 566.45)	-0.72(-0.75 to -0.69)	<0.001
Kiribati	367.88 (256.51 to 523.11)	323.66 (220.37 to 473.35)	-0.42(-0.42 to -0.41)	<0.001
Kuwait	403.27 (283.3 to 567.51)	335.15 (230.14 to 482.05)	-0.62(-0.64 to -0.61)	<0.001
Kyrgyzstan	305.29 (213.18 to 435.39)	275.45 (190.22 to 401.43)	-0.31(-0.33 to -0.29)	<0.001
Lao People's Democratic Republic	264.39 (187.87 to 371.68)	222.79 (152.72 to 322.37)	-0.51(-0.53 to -0.5)	<0.001
Latvia	258.04 (169.79 to 378.4)	238.28 (153.7 to 353.24)	-0.17(-0.21 to -0.14)	<0.001
Lebanon	468.21 (334 to 650.83)	368.43 (260.39 to 524.65)	-0.76(-0.77 to -0.75)	<0.001
Lesotho	523.03 (359.47 to 738.75)	443.42 (300.49 to 652.38)	-0.54(-0.55 to -0.54)	<0.001

Liberia	447.32 (323.6 to 622.57)	365.78 (251.41 to 518.91)	-0.66(-0.68 to -0.63)	<0.001
Libya	513.12 (366.77 to 708.54)	393.8 (278.17 to 551.28)	-0.82(-0.85 to -0.79)	<0.001
Lithuania	248.71 (167.93 to 361.87)	226.96 (149.75 to 337.91)	-0.3(-0.31 to -0.3)	<0.001
Luxembourg	166.5 (109.61 to 245.3)	151.67 (97.26 to 226.7)	-0.29(-0.31 to -0.28)	<0.001
Madagascar	332.51 (229.38 to 473.8)	289.37 (196.37 to 416.98)	-0.44(-0.46 to -0.43)	<0.001
Malawi	416.87 (303.72 to 561.51)	323.89 (231.37 to 446.84)	-0.79(-0.82 to -0.75)	<0.001
Malaysia	408.01 (295.82 to 546.18)	283.46 (202.76 to 391.87)	-1.19(-1.22 to -1.16)	<0.001
Maldives	356.05 (253.05 to 491.77)	263.43 (182.77 to 383.91)	-0.99(-1.01 to -0.97)	<0.001
Mali	451.82 (326.03 to 610.11)	443.58 (321.14 to 611.28)	-0.05(-0.08 to -0.02)	0.002
Malta	170.81 (112.81 to 251.64)	155.16 (99.67 to 232.63)	-0.31(-0.31 to -0.3)	<0.001
Marshall Islands	364.17 (253.25 to 523.15)	300.21 (201.77 to 445.72)	-0.61(-0.63 to -0.6)	<0.001
Mauritania	513.37 (348.17 to 751.72)	441.67 (290.49 to 659.86)	-0.46(-0.5 to -0.42)	<0.001
Mauritius	357.16 (255.47 to 502.67)	281.53 (195.89 to 401.6)	-0.71(-0.74 to -0.67)	<0.001
Mexico	378.24 (269.38 to 525.27)	328.7 (222.15 to 479.52)	-0.24(-0.36 to -0.12)	0.001
Micronesia (Federated States of)	365.89 (256.21 to 525.91)	309.05 (206.78 to 451.75)	-0.54(-0.55 to -0.54)	<0.001
Monaco	161.5 (105.29 to 240.85)	152.66 (97.82 to 228.81)	-0.18(-0.19 to -0.18)	<0.001
Mongolia	390.64 (275.75 to 549.68)	328.28 (229.19 to 473.31)	-0.59(-0.61 to -0.56)	<0.001
Montenegro	161.99 (104.43 to 243.46)	154.32 (98.02 to 230.88)	-0.17(-0.19 to -0.15)	<0.001
Morocco	431.56 (299.36 to 613.62)	368.03 (246.97 to 542.14)	-0.46(-0.49 to -0.43)	<0.001
Mozambique	416.7 (296.29 to 572.58)	393.2 (283.56 to 542.9)	-0.2(-0.23 to -0.17)	<0.001
Myanmar	530.49 (381.64 to 708.72)	384.91 (275.43 to 530.83)	-1.09(-1.14 to -1.02)	<0.001
Namibia	466.78 (323.47 to 671.57)	385.71 (255.88 to 568.52)	-0.61(-0.67 to -0.56)	<0.001
Nauru	344.63 (236.25 to 498.52)	304.77 (203.36 to 448.8)	-0.4(-0.41 to -0.38)	<0.001
Nepal	552.03 (384.72 to 780.48)	508.6 (328.23 to 774.66)	-0.2(-0.33 to 0.01)	0.055
Netherlands	151.62 (97.35 to 227.01)	141.39 (88.55 to 213.28)	-0.22(-0.23 to -0.21)	<0.001
New Zealand	179.08 (112.97 to 264.94)	167.15 (103.69 to 253.08)	-0.21(-0.23 to -0.2)	<0.001

Nicaragua	451.2 (316.05 to 634.45)	362.23 (246.06 to 521.9)	-0.71(-0.72 to -0.71)	<0.001
Niger	495.41 (341.43 to 705.92)	553.78 (384.55 to 797.49)	0.21(0.13 to 0.29)	<0.001
Nigeria	561.34 (404.48 to 780.75)	509.1 (359.62 to 717.61)	-0.35(-0.37 to -0.33)	<0.001
Niue	336.43 (230.45 to 489.06)	283.75 (184.89 to 417.61)	-0.54(-0.56 to -0.53)	<0.001
North Macedonia	172.08 (113.7 to 254)	156.52 (100.65 to 237.35)	-0.32(-0.34 to -0.3)	<0.001
Northern Mariana Islands	306.97 (205.13 to 446.1)	280.38 (183.91 to 420.14)	-0.26(-0.28 to -0.24)	<0.001
Norway	160.84 (101.57 to 244.45)	154.96 (96.33 to 235.15)	-0.13(-0.13 to -0.12)	<0.001
Oman	577.44 (410.72 to 796.79)	524.27 (367.85 to 737.39)	-0.31(-0.35 to -0.26)	<0.001
Pakistan	629.92 (457.11 to 854.58)	589.4 (427.15 to 802.31)	-0.25(-0.3 to -0.19)	<0.001
Palau	323.87 (221.26 to 467.87)	280.83 (184.13 to 419.28)	-0.46(-0.47 to -0.44)	<0.001
Palestine	486.34 (348.85 to 671.59)	363.27 (252.79 to 515.84)	-0.94(-0.96 to -0.93)	<0.001
Panama	448.2 (309.86 to 635.14)	369.5 (253.93 to 534.5)	-0.6(-0.63 to -0.58)	<0.001
Papua New Guinea	453.46 (318.74 to 636.52)	402.62 (277.48 to 574.82)	-0.52(-0.58 to -0.46)	<0.001
Paraguay	473.56 (327.29 to 674.98)	386.77 (252.45 to 565.6)	-0.57(-0.6 to -0.54)	<0.001
Peru	547.22 (381.27 to 770.7)	433.66 (292.31 to 626.48)	-0.8(-0.84 to -0.75)	<0.001
Philippines	491.39 (328.89 to 717.85)	450.51 (295.89 to 673.48)	-0.26(-0.27 to -0.25)	<0.001
Poland	174.97 (112.4 to 264.65)	166.73 (106.57 to 253.92)	-0.17(-0.18 to -0.16)	<0.001
Portugal	177.7 (119.5 to 262.35)	157.62 (102.17 to 236.29)	-0.37(-0.38 to -0.37)	<0.001
Puerto Rico	282.08 (182.47 to 418.08)	246.5 (153.39 to 374.34)	-0.44(-0.45 to -0.44)	<0.001
Qatar	464.38 (332.1 to 644.93)	348.37 (241.73 to 494.07)	-0.91(-0.93 to -0.9)	<0.001
Republic of Korea	188.77 (125.24 to 273.49)	159.3 (101.03 to 242.84)	-0.53(-0.56 to -0.5)	<0.001
Republic of Moldova	316.57 (220.69 to 455.73)	294.89 (200.18 to 427.8)	-0.19(-0.23 to -0.16)	<0.001
Romania	173.38 (114.03 to 257.06)	160.17 (102.95 to 241.75)	-0.26(-0.28 to -0.25)	<0.001
Russian Federation	340.2 (220.37 to 508.16)	345.3 (210.9 to 542.12)	0.14(0.03 to 0.26)	0.017
Rwanda	310.66 (214 to 441.93)	253.89 (167.21 to 375.8)	-0.68(-0.7 to -0.64)	<0.001
Saint Kitts and Nevis	305.8 (206.5 to 441.83)	256.56 (164.33 to 380.98)	-0.56(-0.57 to -0.55)	<0.001

Saint Lucia	304.92 (204.28 to 447.14)	256.7 (163.03 to 387.33)	-0.56(-0.57 to -0.55)	<0.001
Saint Vincent and the Grenadines	313.94 (213.29 to 459.09)	265.72 (171.88 to 391.13)	-0.52(-0.53 to -0.51)	<0.001
Samoa	343.41 (234.25 to 498.66)	298.07 (196.44 to 439.95)	-0.45(-0.46 to -0.44)	<0.001
San Marino	161.05 (105.76 to 239.93)	152.73 (97.51 to 227.04)	-0.17(-0.18 to -0.17)	<0.001
Sao Tome and Principe	469.2 (332.23 to 660.34)	376.81 (258.55 to 535.5)	-0.72(-0.73 to -0.71)	<0.001
Saudi Arabia	858.14 (622.34 to 1140.97)	526.97 (378.58 to 721.31)	-1.59(-1.63 to -1.56)	<0.001
Senegal	502.3 (362.97 to 685.4)	408.12 (294.2 to 575.89)	-0.61(-0.71 to -0.51)	<0.001
Serbia	170.26 (111.08 to 253.46)	156.22 (101.14 to 236.35)	-0.31(-0.36 to -0.26)	<0.001
Seychelles	369.48 (265.24 to 522.27)	294.36 (204.04 to 419.74)	-0.73(-0.74 to -0.71)	<0.001
Sierra Leone	404.2 (285.88 to 569.66)	357.45 (247.36 to 511.61)	-0.42(-0.45 to -0.38)	<0.001
Singapore	200.14 (132.64 to 295.43)	182.52 (119.33 to 270.56)	-0.26(-0.28 to -0.25)	<0.001
Slovakia	153.77 (104.8 to 222.27)	141.08 (93.39 to 207.34)	-0.22(-0.25 to -0.19)	<0.001
Slovenia	171.43 (108.48 to 260.78)	163.25 (102.51 to 250.27)	-0.11(-0.13 to -0.09)	<0.001
Solomon Islands	383 (267.28 to 546.07)	333.04 (227.81 to 486.37)	-0.44(-0.45 to -0.43)	<0.001
Somalia	334.93 (231.7 to 468.53)	317.46 (220.23 to 447.1)	-0.18(-0.23 to -0.14)	<0.001
South Africa	546.16 (349.13 to 823.34)	500.1 (308.7 to 778.18)	-0.16(-0.3 to 0.1)	0.187
South Sudan	493.55 (352.71 to 676.45)	395.92 (282.13 to 554.64)	-0.77(-0.81 to -0.73)	<0.001
Spain	287.4 (202.6 to 406.47)	260.22 (181.01 to 370.64)	-0.28(-0.32 to -0.24)	<0.001
Sri Lanka	383.14 (271.27 to 543.09)	315.4 (216.29 to 457.24)	-0.56(-0.63 to -0.5)	<0.001
Sudan	581.74 (421.48 to 794.43)	412.76 (293.36 to 575.62)	-1.01(-1.03 to -0.98)	<0.001
Suriname	363.34 (250.92 to 520.85)	309.92 (210.25 to 448.16)	-0.54(-0.56 to -0.52)	<0.001
Sweden	120.14 (72.75 to 189.35)	118.21 (71.49 to 187.69)	-0.05(-0.07 to -0.02)	0.002
Switzerland	158.31 (103.48 to 234.65)	149.93 (95.48 to 226.21)	-0.17(-0.18 to -0.17)	<0.001
Syrian Arab Republic	495.18 (359.2 to 673.03)	374.35 (265.16 to 522.64)	-0.86(-0.88 to -0.84)	<0.001
Taiwan (Province of China)	182.59 (97.19 to 315)	174.59 (91.8 to 307.37)	-0.14(-0.15 to -0.13)	<0.001
Tajikistan	344.31 (240.43 to 490.72)	314.52 (218.66 to 454.18)	-0.28(-0.29 to -0.26)	<0.001

Thailand	387.81 (275.51 to 542.99)	295.41 (202.48 to 429.89)	-0.91(-0.97 to -0.86)	<0.001
Timor-Leste	623.24 (450.78 to 837.72)	458.08 (330.31 to 628.73)	-0.81(-0.91 to -0.71)	<0.001
Togo	384.58 (267.57 to 545.71)	395.74 (274.6 to 560.37)	0.03(-0.01 to 0.07)	0.172
Tokelau	359.12 (247.23 to 512.95)	295.66 (194.13 to 432.82)	-0.62(-0.63 to -0.61)	<0.001
Tonga	281.31 (185.92 to 418.2)	243.25 (152.61 to 373.43)	-0.46(-0.48 to -0.44)	<0.001
Trinidad and Tobago	290.32 (190.41 to 429.71)	246.31 (155.01 to 376.04)	-0.56(-0.59 to -0.53)	<0.001
Tunisia	460.09 (332.38 to 634.24)	331.61 (231.65 to 474.24)	-0.98(-1.01 to -0.94)	<0.001
Turkey	344.48 (241.97 to 481.33)	300.8 (195.82 to 452.1)	-0.48(-0.56 to -0.4)	<0.001
Turkmenistan	371.94 (257.26 to 535.78)	320.23 (220.6 to 462.99)	-0.54(-0.58 to -0.5)	<0.001
Tuvalu	380.04 (263.89 to 543.06)	309.59 (208.77 to 452.53)	-0.65(-0.66 to -0.63)	<0.001
Uganda	272.82 (187.42 to 387.49)	269.07 (181.59 to 389.68)	-0.03(-0.05 to -0.02)	<0.001
Ukraine	255.87 (174.61 to 371.03)	240.82 (161.16 to 355.01)	-0.15(-0.19 to -0.11)	<0.001
United Arab Emirates	439.91 (312.81 to 613.17)	335.47 (234.26 to 481.78)	-0.8(-0.84 to -0.76)	<0.001
United Kingdom	182.3 (118.51 to 269.18)	169.11 (107.85 to 253.66)	-0.26(-0.28 to -0.25)	<0.001
United Republic of Tanzania	482.6 (348.99 to 661.41)	402.18 (289.01 to 559.81)	-0.62(-0.73 to -0.5)	<0.001
United States of America	133.41 (84.62 to 202.48)	135.02 (84.34 to 208.14)	0.17(0 to 0.47)	0.045
United States Virgin Islands	288.8 (190.25 to 425.95)	253.76 (159.19 to 380.41)	-0.43(-0.44 to -0.41)	<0.001
Uruguay	188.6 (120.1 to 281.7)	178.13 (110.76 to 270.68)	-0.12(-0.17 to -0.08)	<0.001
Uzbekistan	336.76 (234.17 to 481.74)	298.62 (205.26 to 435.98)	-0.4(-0.41 to -0.39)	<0.001
Vanuatu	299.86 (201.29 to 440.03)	273.39 (175.59 to 408.87)	-0.29(-0.3 to -0.29)	<0.001
Venezuela (Bolivarian Republic of)	387.6 (266.8 to 554.05)	338.75 (227.91 to 495.08)	-0.46(-0.51 to -0.4)	<0.001
Viet Nam	396.59 (285.37 to 540.47)	292.73 (210.46 to 407.63)	-1.02(-1.04 to -1)	<0.001
Yemen	405.8 (289.25 to 563)	408.93 (291.5 to 566.12)	0.01(-0.02 to 0.04)	0.739
Zambia	357.98 (253.65 to 502.19)	298.53 (206.24 to 428.77)	-0.53(-0.59 to -0.48)	<0.001
Zimbabwe	433.13 (306.12 to 606.69)	381.42 (262.86 to 547.51)	-0.28(-0.35 to -0.22)	<0.001

Abbreviations: ASR, age-standardized rate; AAPC, average annual percent change; UI, uncertainty interval; DALY, disability-adjusted life year.

Supplementary table 4.. BAPC model predictions of blindness and vision loss and its specific causes by gender subgroups up to 2050.

Measure	Location	Year	Sex	Cause	Number	ASR
Prevalence	Global	2050	Male	Blindness and vision loss	1400765077.44(0 to 3531077797.8)	17060.01(-4447.2 to 38567.23)
Prevalence	Global	2050	Female	Blindness and vision loss	1743449809.84(0 to 4375976101.05)	19791.34(-5127.36 to 44710.05)
Prevalence	Global	2050	Both	Blindness and vision loss	3144214887.29(0 to 7907053898.85)	22796.63(0 to 57378.14)
Prevalence	Global	2050	Male	Age-related macular degeneration	6077802.89(297112.91 to 11873910.8)	66.85(6.82 to 126.88)
Prevalence	Global	2050	Female	Age-related macular degeneration	9314099.39(1310187.15 to 17322175.1)	84.9(15.93 to 153.86)
Prevalence	Global	2050	Both	Age-related macular degeneration	15391902.29(1607300.07 to 29196085.91)	84.37(8.43 to 160.48)
Prevalence	Global	2050	Male	Cataract	90888747.32(18054714.7 to 163730315.33)	1040.33(265.48 to 1815.18)
Prevalence	Global	2050	Female	Cataract	130879199.02(22612153.36 to 239160594.62)	1219.67(278.97 to 2160.38)
Prevalence	Global	2050	Both	Cataract	221767946.34(40666868.06 to 402890909.95)	1227.68(224.25 to 2231.37)
Prevalence	Global	2050	Male	Glaucoma	5878937.59(867902.87 to 10890995.06)	64.45(13.14 to 115.77)
Prevalence	Global	2050	Female	Glaucoma	6008752.23(209063.24 to 11824159.4)	51.82(5.66 to 97.97)
Prevalence	Global	2050	Both	Glaucoma	11887689.82(1076966.11 to 22715154.46)	63.19(5.56 to 120.97)
Prevalence	Global	2050	Male	Near vision loss	1423929179.58(0 to 4305587245.26)	15756.52(-8194.09 to 39707.12)
Prevalence	Global	2050	Female	Near vision loss	1748620183.89(0 to 5086721782.47)	18478.54(-8711.64 to 45668.72)
Prevalence	Global	2050	Both	Near vision loss	3172549363.47(0 to 9392309027.73)	23133.53(0 to 68530.15)
Prevalence	Global	2050	Male	Refraction disorders	101640536.41(30399340.86 to 172881731.95)	1603.29(526.3 to 2680.27)
Prevalence	Global	2050	Female	Refraction disorders	121115931.17(37071825.58 to 205160036.77)	1814.19(607.36 to 3021.02)
Prevalence	Global	2050	Both	Refraction disorders	222756467.58(67471166.44 to 378041768.72)	1815.17(534.61 to 3095.74)
Prevalence	Global	2050	Male	Other vision loss	26336404.92(10601771.46 to 42071038.38)	345.56(144.92 to 546.19)
Prevalence	Global	2050	Female	Other vision loss	36086683.79(13925195.22 to 58248172.36)	405.13(164.29 to 645.97)
Prevalence	Global	2050	Both	Other vision loss	62423088.71(24526966.68 to 100319210.75)	394.7(153.03 to 636.36)
DALYs (Disability-Adjusted Life Years)	Global	2050	Male	Blindness and vision loss	25100263.1(737606.07 to 49503666.37)	325.77(35.36 to 616.17)
DALYs (Disability-Adjusted Life Years)	Global	2050	Female	Blindness and vision loss	32626639.15(0 to 66472653.41)	373.47(20.43 to 726.5)

DALYs (Disability-Adjusted Life Years)	Global	2050	Both	Blindness and vision loss	57726902.25(737606.07 to 115976319.78)	392.12(4.93 to 788.69)
DALYs (Disability-Adjusted Life Years)	Global	2050	Male	Age-related macular degeneration	539595.08(0 to 1215681.12)	5.58(-0.6 to 11.76)
DALYs (Disability-Adjusted Life Years)	Global	2050	Female	Age-related macular degeneration	1012984.83(0 to 2408005.13)	8.21(-1.56 to 17.98)
DALYs (Disability-Adjusted Life Years)	Global	2050	Both	Age-related macular degeneration	1552579.91(0 to 3623686.25)	8.41(0 to 19.67)
DALYs (Disability-Adjusted Life Years)	Global	2050	Male	Cataract	4673691.47(1099244.27 to 8249011.52)	54.81(15.55 to 94.06)
DALYs (Disability-Adjusted Life Years)	Global	2050	Female	Cataract	7110496.87(1550998.3 to 12672073.26)	68.4(18.08 to 118.72)
DALYs (Disability-Adjusted Life Years)	Global	2050	Both	Cataract	11784188.34(2650242.57 to 20921084.78)	66.68(14.87 to 118.53)
DALYs (Disability-Adjusted Life Years)	Global	2050	Male	Glaucoma	616659.12(162408.66 to 1070963.13)	6.84(2.06 to 11.63)
DALYs (Disability-Adjusted Life Years)	Global	2050	Female	Glaucoma	593105.13(128303.62 to 1057959.62)	5.21(1.34 to 9.09)
DALYs (Disability-Adjusted Life Years)	Global	2050	Both	Glaucoma	1209764.24(290712.28 to 2128922.75)	6.37(1.5 to 11.24)
DALYs (Disability-Adjusted Life Years)	Global	2050	Male	Near vision loss	14260098.86(0 to 43662305.61)	157.56(-84.91 to 400.02)
DALYs (Disability-Adjusted Life Years)	Global	2050	Female	Near vision loss	17069324.59(0 to 49844832.13)	181.73(-86.66 to 450.13)
DALYs (Disability-Adjusted Life Years)	Global	2050	Both	Near vision loss	31329423.45(0 to 93507137.75)	230.32(0 to 687.9)
DALYs (Disability-Adjusted Life Years)	Global	2050	Male	Refraction disorders	4016419.73(1310811.21 to 6722028.25)	62.43(22.05 to 102.8)
DALYs (Disability-Adjusted Life Years)	Global	2050	Female	Refraction disorders	4667660.7(1471468.67 to 7863852.73)	68.01(23.27 to 112.76)
DALYs (Disability-Adjusted Life Years)	Global	2050	Both	Refraction disorders	8684080.43(2782279.88 to 14585880.98)	69.11(21.55 to 116.66)
DALYs (Disability-Adjusted Life Years)	Global	2050	Male	Other vision loss	1983518.84(749793.58 to 3217244.1)	26.8(10.66 to 42.94)
DALYs (Disability-Adjusted Life Years)	Global	2050	Female	Other vision loss	2442065.08(876353.11 to 4007777.05)	28.01(10.59 to 45.44)
DALYs (Disability-Adjusted Life Years)	Global	2050	Both	Other vision loss	4425583.92(1626146.69 to 7225021.15)	28.87(10.48 to 47.27)

Abbreviations: ASR, age-standardized rate; BAPC, Bayesian Age-Period-Cohort.

Supplementary figures

Supplementary Figure 1. Global maps of AAPC results for blindness and vision loss in ASPR and ASR of DALY across 204 countries.

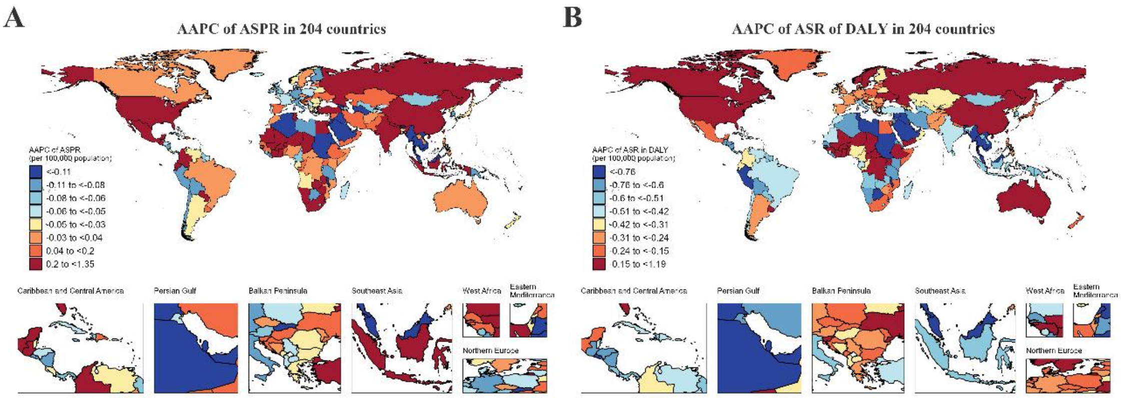

Notes: Supplementary figure 1A shows the global map of AAPC results for ASPR from 1990 to 2021 across 204 countries. Supplementary figure 1B shows the global map of AAPC results for ASR of DALY from 1990 to 2021 across 204 countries.

Abbreviations: ASR, age-standardized rate; ASPR, age-standardized prevalence rate; AAPC, average annual percent change; DALY, disability adjusted life year.

Supplementary Figure 2. Trend analysis of blindness and vision loss in ASPR and ASR of DALY by gender subgroups.

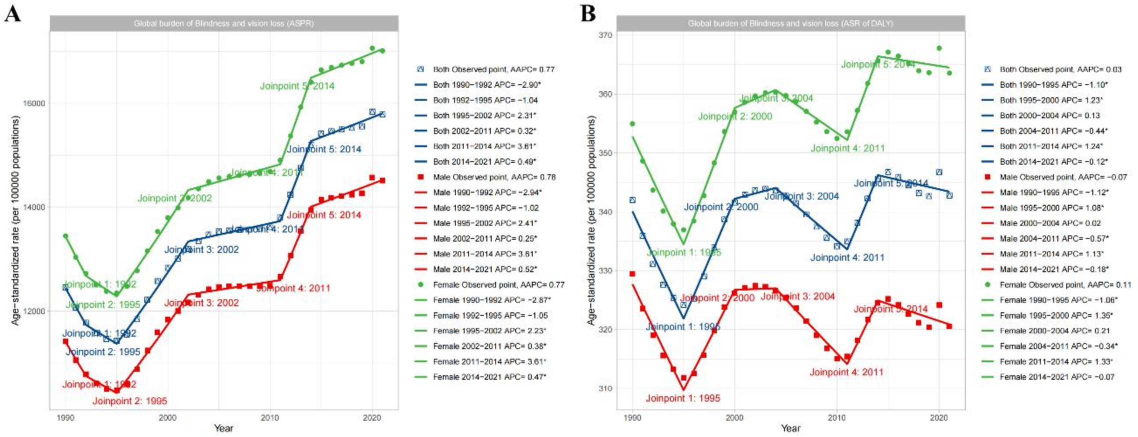

Notes: Supplementary Figure 2A shows the trend analysis results for blindness and vision loss by gender subgroups in ASPR from 1990 to 2021; Supplementary Figure 2B shows the trend analysis results for blindness and vision loss by gender subgroups in ASR of DALY from 1990 to 2021.

Abbreviations: ASR, age-standardized rate; ASPR, age-standardized prevalence rate; AAPC, average annual percent change; DALY, disability adjusted life year; APC, annual percent change.

Supplementary Figure 3. Trend analysis of ASR of DALY for blindness and vision loss caused by different conditions from 1990 to 2021.

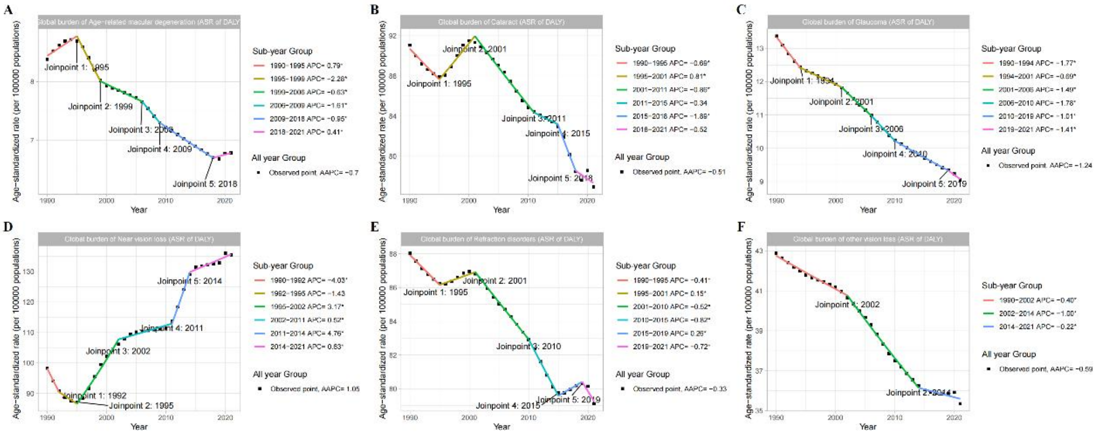

Notes: Supplementary Figure 3A, B, C, D, E, and F correspond to the ASR of DALY trend results for blindness and vision loss caused by age-related macular degeneration, cataract, glaucoma, near vision loss, refraction disorders, and other vision loss, respectively.

Abbreviations: ASR, age-standardized rate; AAPC, average annual percent change; DALY, Disability Adjusted Life Year; APC, annual percent change.

Supplementary Figure 4. Correlation analysis results between ASPR and ASR of DALY and SDI levels in 204 countries globally in 2021.

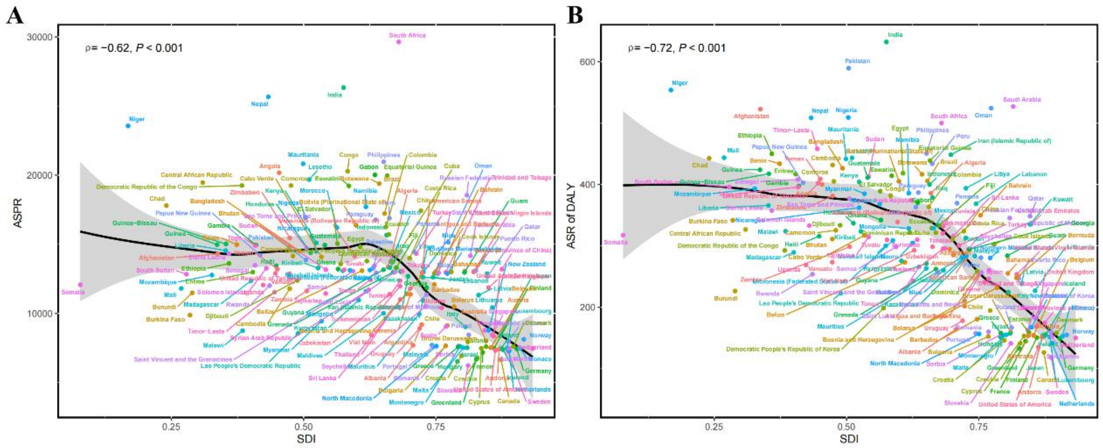

Notes: Supplementary Figure 4A shows the correlation analysis results between ASPR and SDI levels in 204 countries globally in 2021; Supplementary Figure 4B shows the correlation analysis results between ASR of DALY and SDI levels in 204 countries globally in 2021.

Abbreviations: ASPR, age-standardized prevalence rate; ASR, age-standardized rate; DALY, Disability Adjusted Life Year; SDI, Socio-demographic Index.

Supplementary Figure 5. Comparison of trends in ASPR and ASR of DALYs for blindness and vision loss and their specific causes across regions with different SDI levels globally.

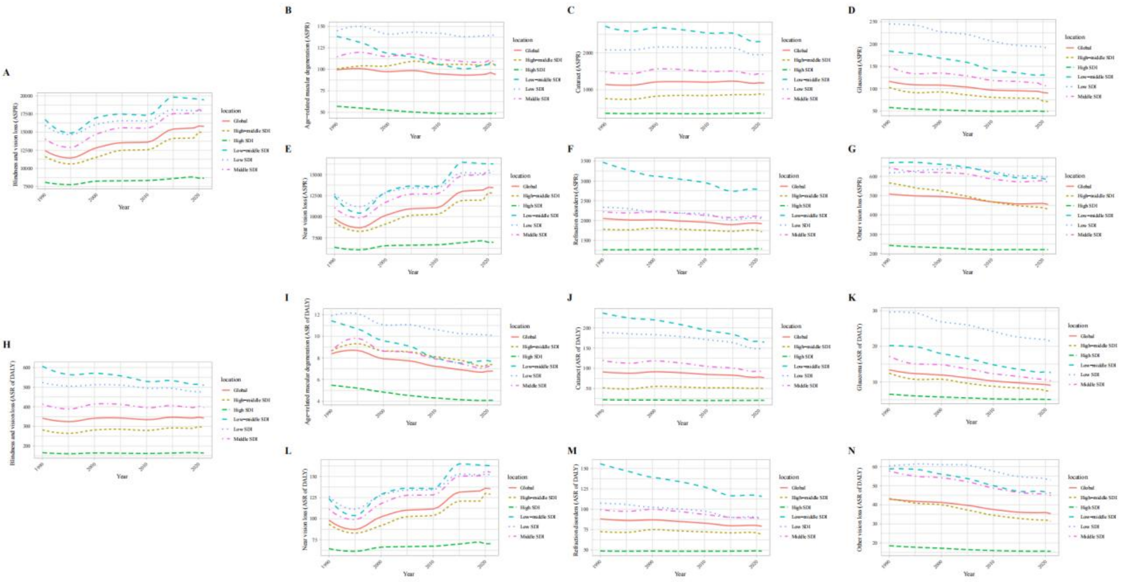

Notes: Supplementary Figure 5A presents the trends in ASPR for blindness and vision loss globally and across the five SDI regions. Supplementary Figures 5B-G illustrate the trends in ASPR for age-related macular degeneration, cataract, glaucoma, near vision loss, refractive disorders, and other vision loss globally and across the five SDI regions, respectively. Supplementary Figure 5H shows the trends in ASR of DALYs for blindness and vision loss globally and across the five SDI regions. Supplementary Figures 5I-N depict the trends in ASR of DALYs for age-related macular degeneration, cataract, glaucoma, near vision loss, refractive disorders, and other vision loss globally and across the five SDI regions, respectively.

Abbreviations: ASPR, age-standardized prevalence rate; ASR, age-standardized rate; DALY, disability adjusted life year.

Supplementary Figure 6. Age subgroup analysis in 2021 showing the proportion of six different causes of blindness and vision loss in the prevalence and DALY number metrics.

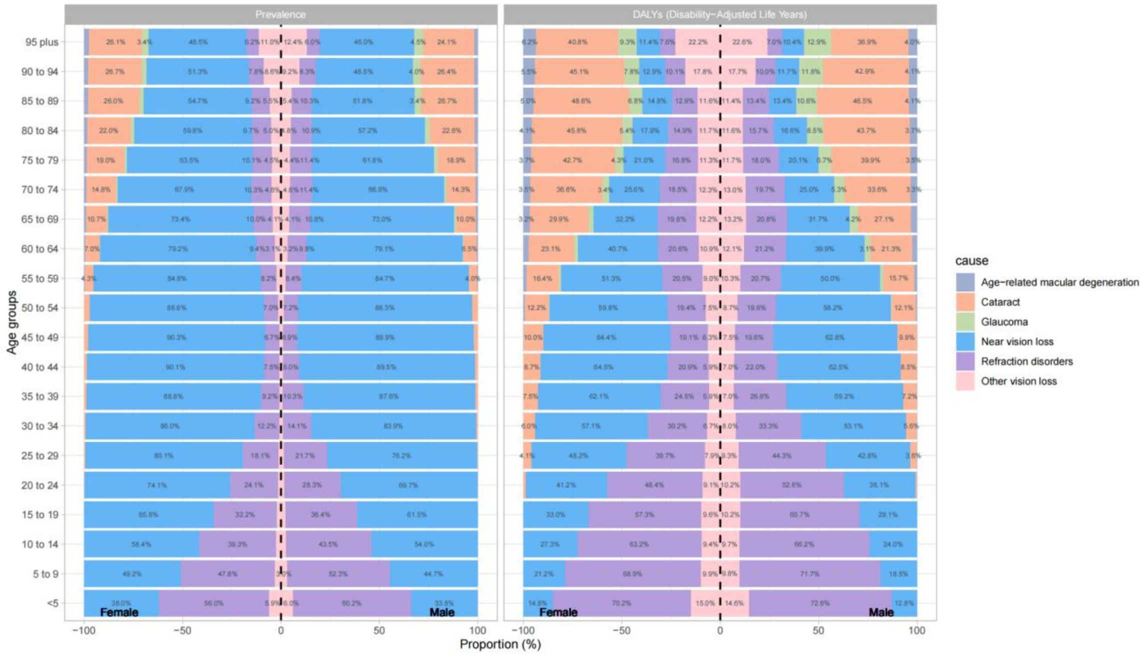

Supplementary Figure 7. Distribution of prevalence and prevalence rates, as well as DALY counts and DALY rates, for blindness and vision loss across different age groups globally in 1990 and 2021.

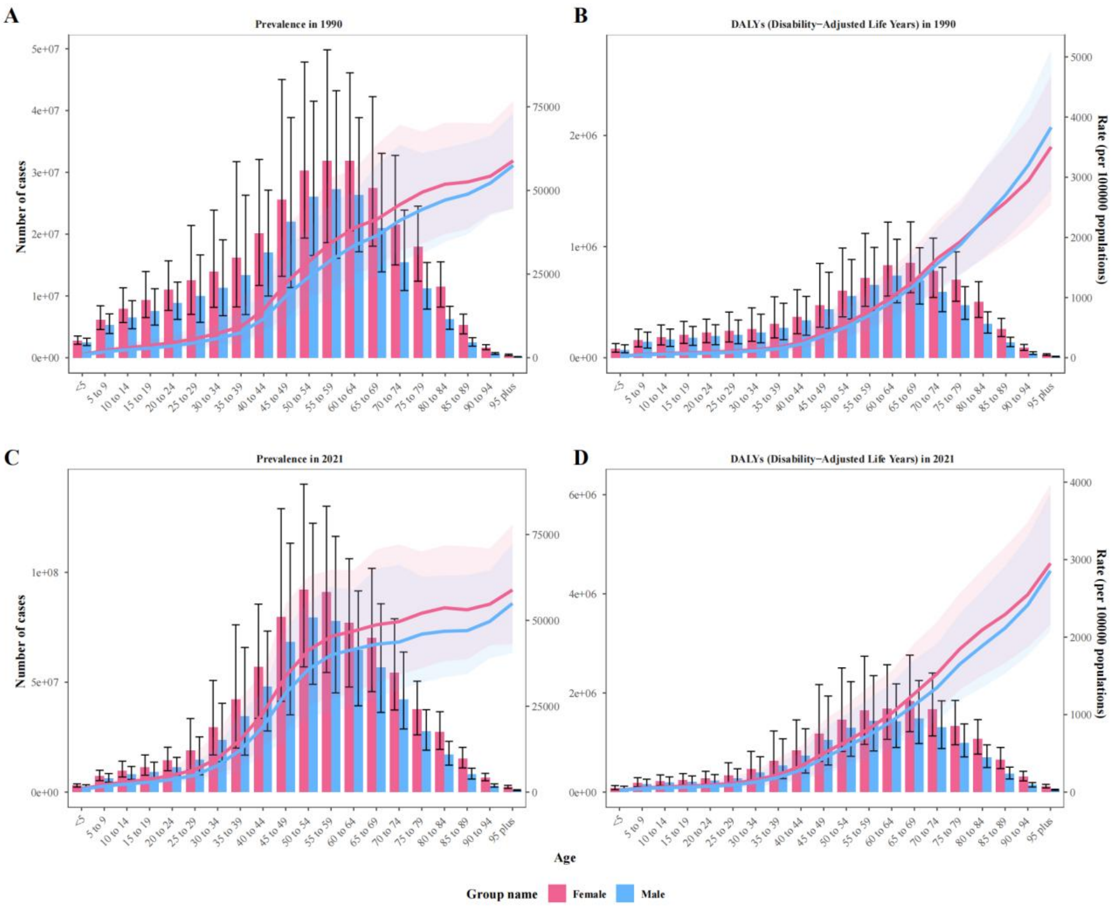

Notes: Supplementary Figures 7A and 6B show dual Y-axis plots of the distribution of prevalence number and prevalence rates, as well as DALY number and DALY rates, for blindness and vision loss across different age groups globally in 1990. Supplementary Figures 7C and 6D present dual Y-axis plots of the same distributions for 2021.

Abbreviations: DALY, disability adjusted life year.

Supplementary Figure 8. Trends in blindness and vision loss prevalence numbers and their causes globally and across 26 regions from 1990 to 2021.

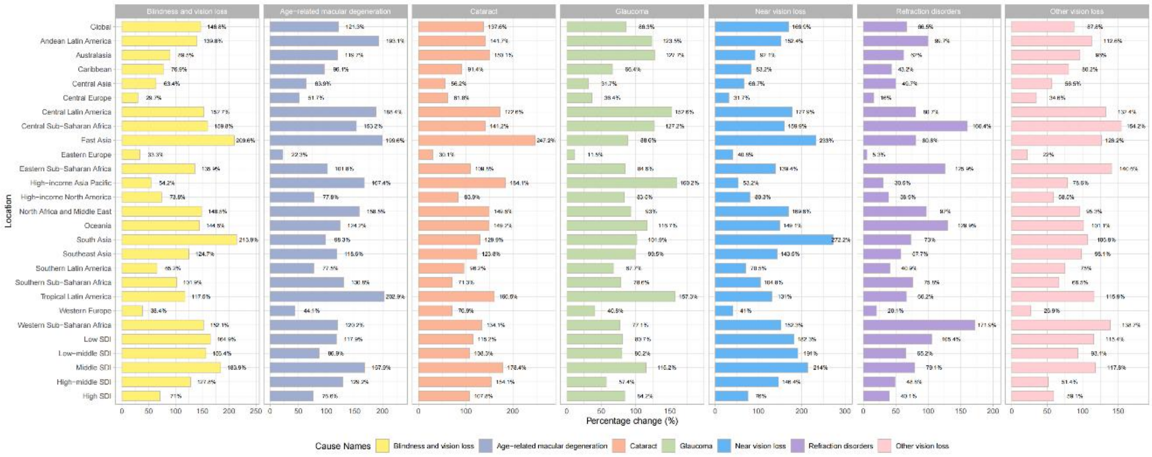

Supplementary Figure 9. Trends in blindness and vision loss ASPR and its causes globally and across 26 regions from 1990 to 2021.

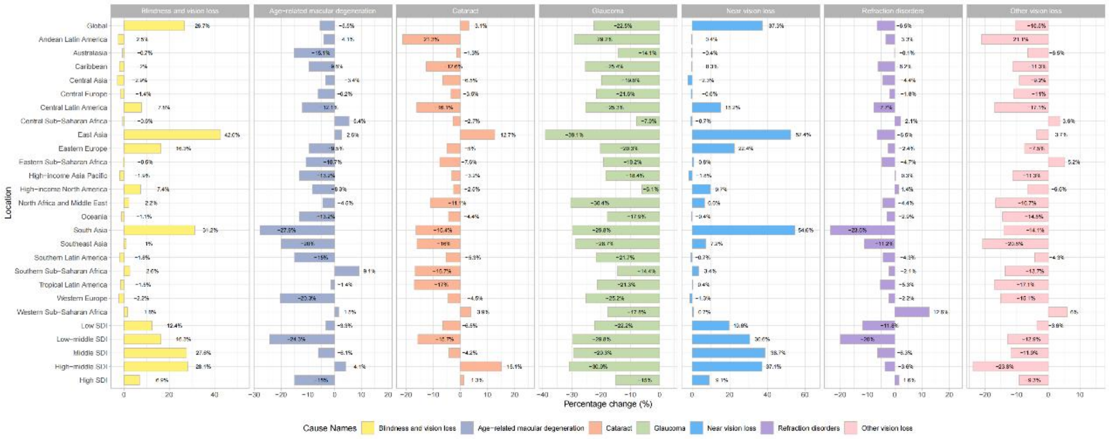

Abbreviations: ASPR, age-standardized prevalence rate.

Supplementary Figure 10. Trends in blindness and vision loss DALY numbers and their causes globally and across 26 regions from 1990 to 2021.

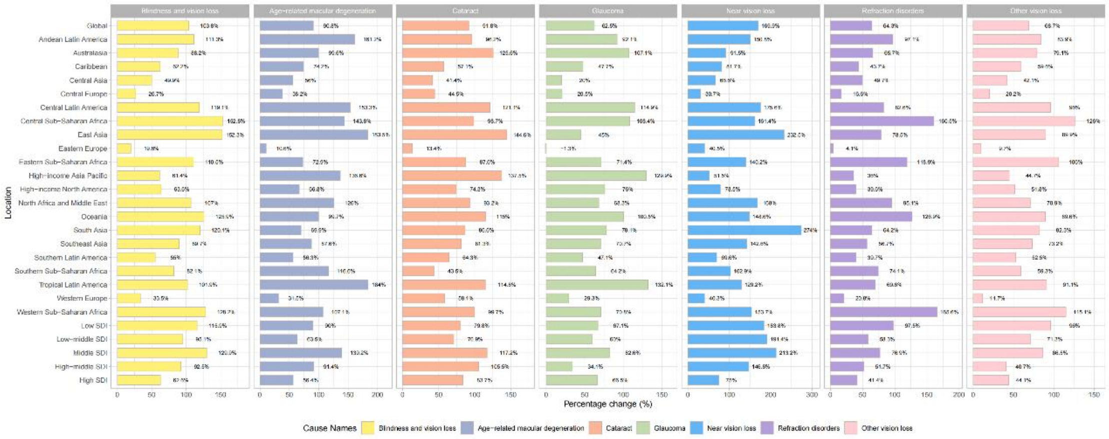

Abbreviations: DALY, disability-adjusted life-year.

Supplementary Figure 11. Trends in blindness and vision loss ASR of DALY and its causes globally and across 26 regions from 1990 to 2021.

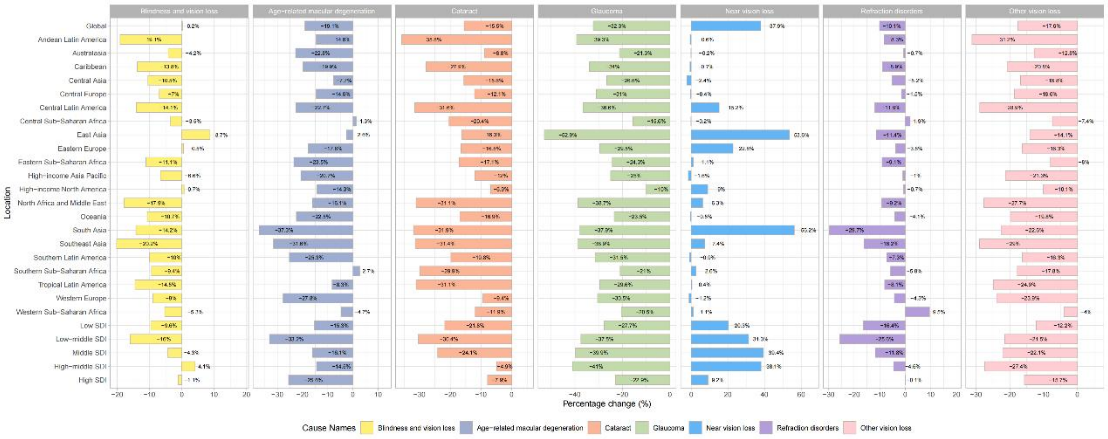

Abbreviations: DALY, disability-adjusted life-year; ASR, age-standardized rate.

Supplementary Figure 12. Back-testing validation of the BAPC model for global blindness and vision loss (2010–2021).

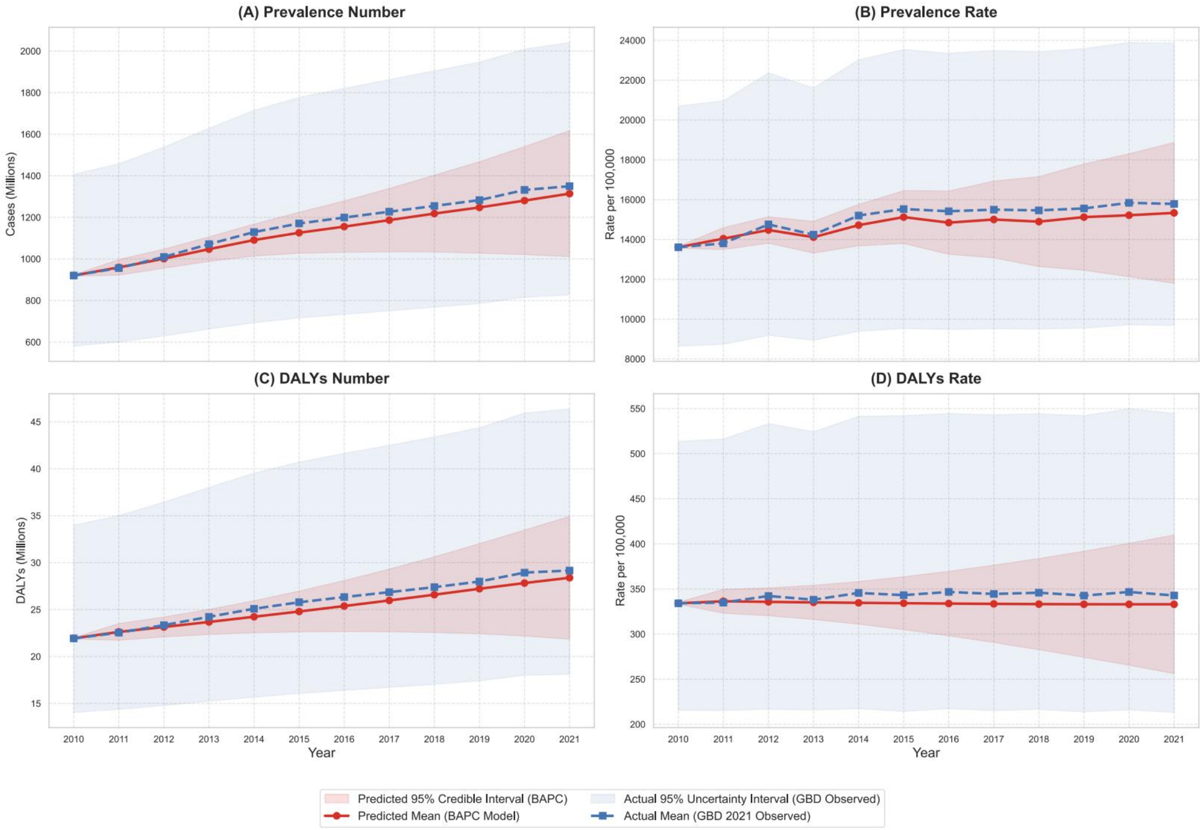

Notes: Panels represent: (A) Prevalence Number (Millions); (B) Prevalence Rate (per 100,000); (C) DALYs Number (Millions); and (D) DALYs Rate (per 100,000). The red solid lines and corresponding shaded areas indicate the predicted means and 95% Credible Intervals (CrIs) from the BAPC model, respectively. The blue dashed lines and shaded areas represent the observed means and 95% Uncertainty Intervals (UIs) from the GBD 2021 study. The high degree of overlap between the predicted trajectories and the observed data confirms the robustness of the second-order random walk (RW2) priors and the model's ability to accurately capture the epidemiological momentum of vision loss.

Supplementary Figure 13. BAPC prediction of blindness and vision loss by gender subgroups up to 2050.

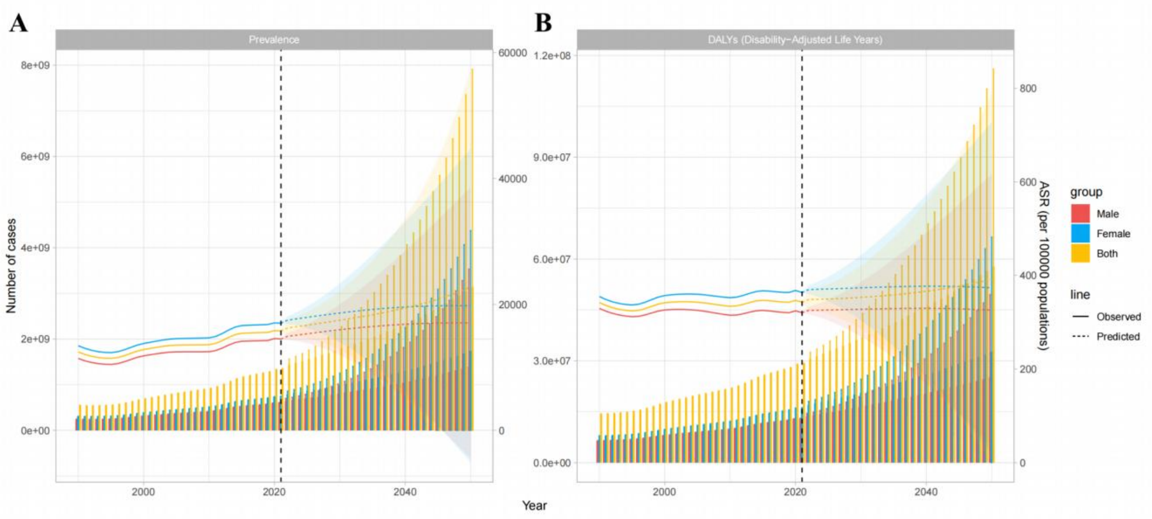

Notes: Supplementary Figure 13A shows the predicted prevalence of blindness and vision loss up to 2050; Supplementary Figure 13B shows the predicted DALY of blindness and vision loss up to 2050.

Abbreviations: BAPC, Bayesian Age-Period-Cohort model; ASR, age-standardized rate; DALY, disability adjusted life year.

Supplementary Figure 14. BAPC prediction of different causes of blindness and vision loss by gender subgroups up to 2050.

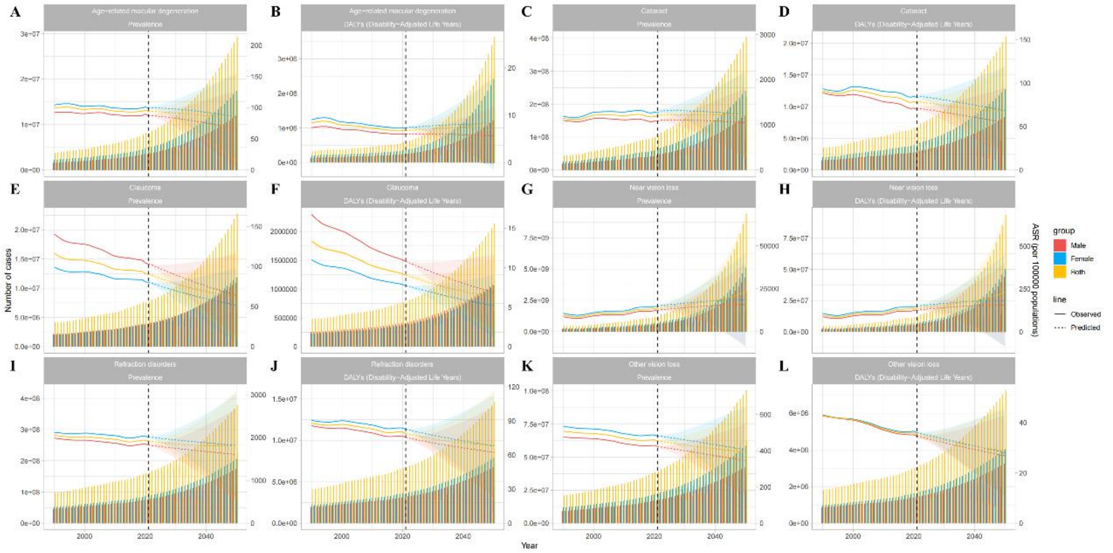

Notes: Supplementary Figures 14A and 14B represent the predicted prevalence and DALY of age-related macular degeneration up to 2050; Supplementary Figures 14C and 14D represent the predicted prevalence and DALY of cataract up to 2050; Supplementary Figures 14E and 14F represent the predicted prevalence and DALY of glaucoma up to 2050; Supplementary Figures 14G and 14H represent the predicted prevalence and DALY of near vision loss up to 2050; Supplementary Figures 14I and 14J represent the predicted prevalence and DALY of refraction disorders up to 2050; Supplementary Figures 14K and 14L represent the predicted prevalence and DALY of other vision loss up to 2050.

Abbreviations: BAPC, Bayesian Age-Period-Cohort model; ASR, age-standardized rate.
